# Effects of antioxidant-rich *foods* on altitude-induced oxidative stress and inflammation in elite endurance athletes: A randomized controlled trial

**DOI:** 10.1371/journal.pone.0217895

**Published:** 2019-06-13

**Authors:** Anu Elisa Koivisto, Thomas Olsen, Ingvild Paur, Gøran Paulsen, Nasser Ezzatkhah Bastani, Ina Garthe, Truls Raastad, Jason Matthews, Rune Blomhoff, Siv Kjølsrud Bøhn

**Affiliations:** 1 Norwegian Olympic Sports Centre, Norwegian Olympic and Paralympic Committee and Confederation of Sports, Oslo, Norway; 2 Department of Nutrition, Institute of Basic Medical Sciences, University of Oslo, Oslo, Norway; 3 Division of Clinical Service, Division of Cancer Medicine, Oslo University Hospital, Oslo, Norway; 4 Department of Physical Performance, Norwegian School of Sport Sciences, Oslo, Norway; 5 Department of Chemistry, Biotechnology and Food Sciences, Norwegian University of Life Sciences, Ås, Norway; Fondazione Toscana Gabriele Monasterio, ITALY

## Abstract

**Background:**

Various altitude training regimes, systematically used to improve oxygen carrying capacity and sports performance, have been associated with increased oxidative stress and inflammation. We investigated whether increased intake of common antioxidant-rich foods attenuates these processes.

**Methods:**

In a randomized controlled trial, 31 elite endurance athletes (23 ± 5 years), ingested antioxidant-rich foods (n = 16), (> doubling their usual intake), or eucaloric control foods (n = 15) during a 3-week altitude training camp (2320 m). Fasting blood and urine samples were collected 7 days pre-altitude, after 5 and 18 days at altitude, and 7 days post-altitude. Change over time was compared between the groups using mixed models for antioxidant capacity [uric acid-free (ferric reducing ability of plasma (FRAP)], oxidative stress (8-epi-PGF_2α_) and inflammatory biomarkers (IFNγ, IL1α, IL1RA, IL1β, IL2, IL5, IL6, IL7, IL10, IL12p70, IL13, IL17, TNFα, MCP-1 and micro-CRP). The cytokine response to a stress-test (VO_2max_ ramp test or 100 m swimming) was assessed at pre- and post-altitude.

**Results:**

FRAP increased more in the antioxidant compared to the control group (p = 0.034). IL13 decreased in the antioxidant group, while increasing in the controls (p = 0.006). A similar trend was seen for IL6 (p = 0.062). A larger decrease in micro-CRP was detected in the antioxidant group compared to controls (β: -0.62, p = 0.02). We found no group differences for the remaining cytokines. 8-epi-PGF_2α_ increased significantly in the whole population (p = 0.033), regardless group allocation. The stress response was significantly larger post-altitude compared with pre-altitude for IL1β, IL6, IL7, IL13, IL12p70 and TNFα, but we found no group differences.

**Conclusions:**

Increased intake of antioxidant-rich foods elevated the antioxidant capacity and attenuated some of the altitude-induced systemic inflammatory biomarkers in elite athletes. The antioxidant intervention had no impact on the altitude-induced oxidative stress or changes in acute cytokine responses to exercise stress-tests.

## Introduction

Several endurance athletes incorporate various hypoxic training modalities to their annual training plan to increase their oxygen carrying capacity and ultimately improve sports performance [1]. The combination of hypoxia and exercise can result in a more pronounced impact on immune system than hypoxia or exercise alone [2–4]. During altitude training camps athletes seem to be at increased risk for immunological disturbances, infections and illness [4–6], which may jeopardize the desired altitude-induced increase in hemoglobin mass [7, 8]. The systemic concentration of several inflammatory cytokines increases following training at altitude illustrating an immune system response to hypoxia [9, 10]. Altitude training is also associated with an elevation in free radical production, reduction in plasma antioxidant capacity and subsequent increase in oxidative stress [11–13]. Both acute [14] and long-term hypoxic exposures [15, 16] augment oxidative stress, and the magnitude of the oxidative stress response seems to depend on the total hypoxic dose (duration and meters above sea level) [17]. Although the underlying mechanisms of hypoxia-induced reactive oxygen species (ROS) overproduction are not entirely clear, reductive stress within the mitochondria, augmented catecholamine production, decreased mitochondria redox potential and xanthine oxidase pathway activation, have previously been suggested [12].

Diets low in antioxidant-rich foods are associated with increased plasma inflammatory mediators and decreased plasma antioxidant concentration in endurance athletes both at rest and following exercise [18, 19]. Whereas, it has been suggested that athletes on a high-antioxidant diet may experience increased protection against training-induced respiratory illness by better maintenance of the pro-oxidant/antioxidant balance, especially at altitude [20]. Several authors have studied the impact of dietary antioxidants on redox balance and biomarkers of oxidative stress and inflammation in well trained athletes, but these have mainly investigated supplements and extracts [21–24], with only a few utilizing a food-based approach to augment antioxidant intake [25–27]. To our knowledge, no previous study has examined the effects of common antioxidant-rich foods on biomarkers of oxidative stress and inflammation during training at altitude in athletes. Given the increasing awareness around the potential negative effects of chronic high dose antioxidant supplementation on training adaptation [28, 29], we chose to apply a food-based approach.

Thus, the aim of the current study was to determine whether increased consumption of foods naturally rich in antioxidants influences biomarkers of systemic oxidative stress and inflammation in response to training at moderate altitude in elite endurance athletes during their general preparation phase. We also aimed at testing whether the antioxidant-rich foods would affect the acute systemic inflammatory stress response to a maximal physical exertion stress-test (VO_2max_ ramp test or 100 m swimming). We conducted a randomized controlled trial to test our hypothesis that increased intake of antioxidant-rich foods would limit these oxidative stress and inflammatory responses to altitude training.

## Methods

### Compliance with ethical standards

This study protocol (S1 Text) was approved by the Norwegian Regional Ethics Committee (REK number 626539) 12^th^ October 2015. All participants provided written informed consent after receiving comprehensive oral and written information about the project protocol following the formal enrollment 12-18^th^ October. After a short delay the study was also registered in Clinical trials (NCT03088891) The registration in clinicaltrials.gov was not a prerequisite for ethical approval in Norway at the time of the project initiation.

### Study design

**Fig 1:**
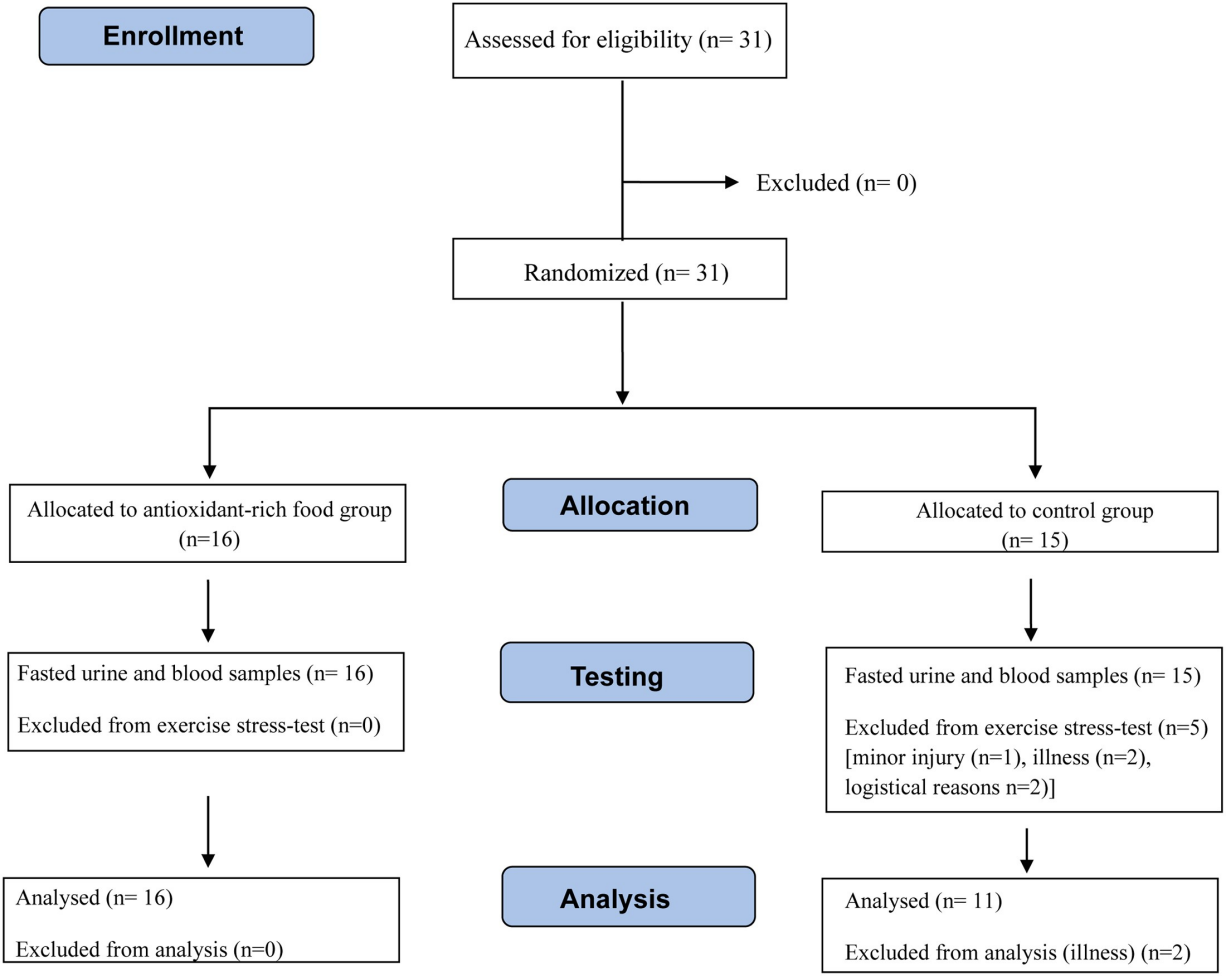
CONSORT flowchart.

**Fig 2:**
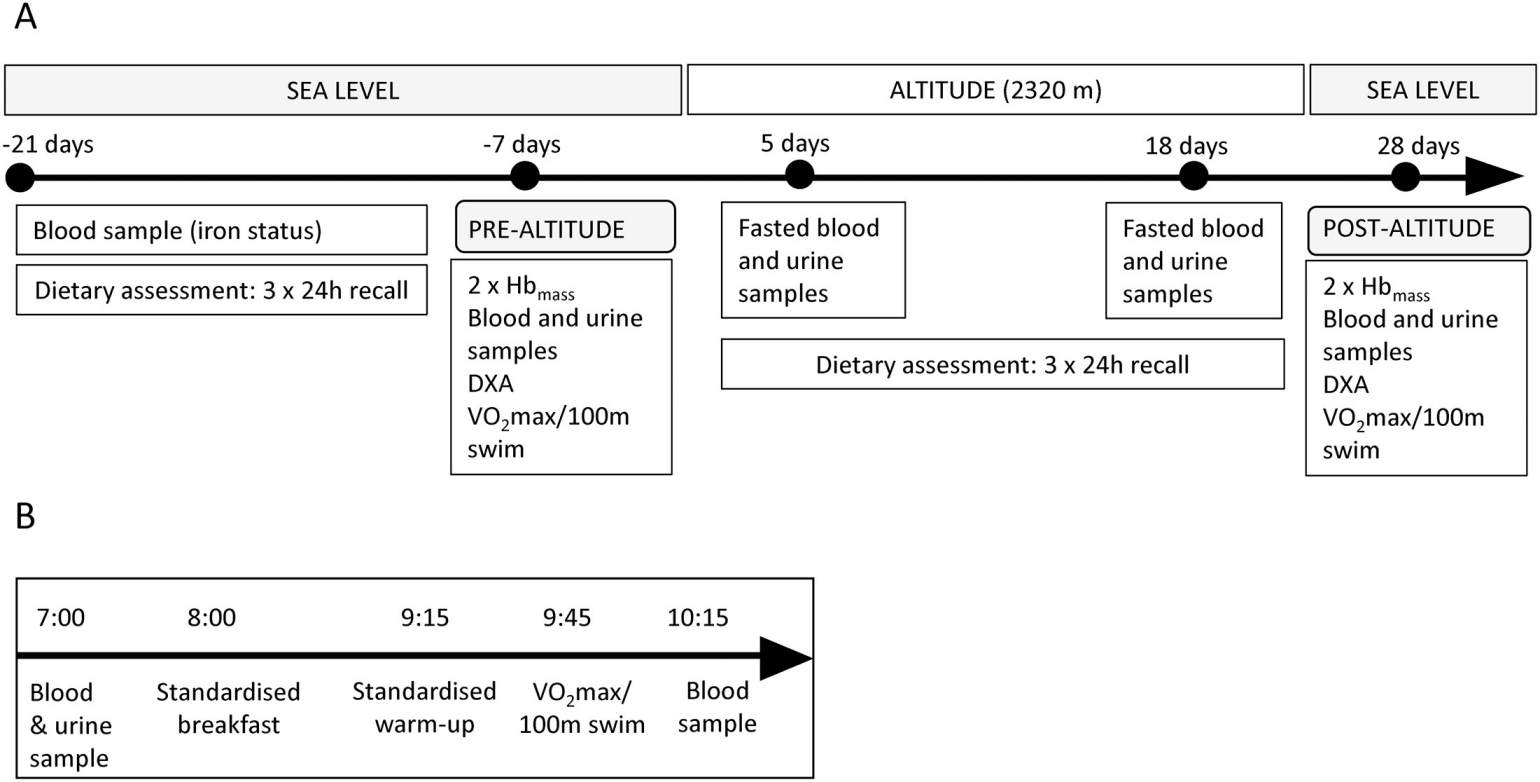
Timeline of the study. A) Timeline of testing before, during and after the three-week altitude training camp (2320m), and B) the setup for the pre-and post-altitude stress-tests (VO_2max_ ramp test or 100 m swimming).

The CONSORT flowchart (Fig 1) shows the outline of the study. This parallel randomized clinical trial with allocation ratio ~1:1 was conducted in October- November 2015, during the athletes’ general preparation phase, less than a year prior to the Rio 2016 Olympic and Paralympic Games. Data and samples before and after the altitude camp were collected at the Norwegian Olympic Sports Centre, Oslo, Norway while data and sample collection during the 3-week altitude camp (21^st^ October–10^th^ November) took place at the High-Altitude Training Centre (Centro di Alto Rendimiento, CAR) in Sierra Nevada, Spain (2320m) (Fig 2). All testing was completed within one week pre-and post-altitude camp for every athlete except for the swimmers. Due to logistical challenges, the swimmers’ stress-tests post altitude were completed thirteen days after return to sea level (on Nov 24^th^). Assessment of iron status three weeks prior to altitude camp (Fig 1) is a general part of the athletes’ regular health monitoring and was used to provide information whether iron supplementation at altitude was required.

### Participants and allocation to interventions

In total, 31 national team athletes from different sports, who attended the yearly altitude training camp arranged by the Norwegian Olympic Sports Centre, were invited to this study (females n = 8, males = 23; Paralympic athletes = 4, Olympic athletes = 27), including seven World Championship medalists (Table 1). All invited athletes agreed to participate. Participants were randomly allocated to receive either antioxidant-rich foods or eucaloric control foods with significantly lower antioxidant content. Each participant was given a random id number prior to randomization. The randomization to intervention or control croup was performed using computer generated random sequence stratified by sport and gender. The researcher who performed the randomization was not involved in the participant enrolment or group allocation. All researchers involved in testing and sample analysis were blinded. During the altitude training camp, athletes followed their respective National teams’ training programs and lived and consumed all their main meals at CAR. The total hypoxic exposure was between 440–480 hours at 2320m above sea level. For practical reasons the pre-and post-altitude tests were conducted over two days [30]. All assessments included in the present paper were conducted on day two (Fig 2B).

**Table 1:** Baseline characteristics.

	Antioxidant group (n = 16)	Controls (n = 15)	*p*^1^
Age (yrs)	23 ± 5	24 ± 5	0.622
Height (cm)	185 ± 8	185 ± 9	0.848
Weight (kg)	81.8 (31.8)	75.9 (38.8)	0.892
VO_2max_ (mL/kg/min)	67.6 (24.2)	66.5 (8.1)	0.428
Training volume (hrs/week)	20.1 ± 6.3	17.0 ± 5.0	0.234
Sex			
*males*	12 (75%)	11 (73%)	1.0^b^
*females*	4 (25%)	4 (27%)	
Able-bodied/disabled athletes			
*Able-bodied athletes*	14 (88%)	13 (87%)	1.0^b^
*Athletes with disabilities*	2 (13%)	2 (13%)	

Values are presented as mean ± standard deviation (SD) or median (range) for non-normally distributed data or count (%).

^1^Indicates difference between groups.

^b^ Fisher’s exact tests were used to compare categorical variables between the groups.

### Dietary intervention

All intervention foods, both for the antioxidant and control group were provided by the Norwegian Olympic Sports Centre, shipped to Spain from Norway, weighed with 1-gram accuracy (Electronic kitchen scale, Page Evolution, Soehnle, Germany), packed and delivered daily at the same time for the whole 3-week period. Study participants in the antioxidant-rich food group received 750 ml fruit-, vegetable- and berry smoothie, 50 g dried berries and fruits, 40 g walnuts, and 40 g dark chocolate (70% cocoa content) daily, while the control group received 220 ml milkshake, 330 ml recovery beverage, 90 g salty and sweet crackers, and 50 g white chocolate. The total antioxidant content of foods, measured with the FRAP method [31], was 21.2 mmol/day and 2.8 mmol/day for antioxidant-rich and control group, respectively. See Koivisto *et al*. [30] for detailed information about the food item selection and the antioxidant content of each food item. The participants were asked to consume the food items (isocaloric, 4.2 MJ or 1000 kcal/day for both groups) between their main meals, thus replacing some of their usual snacks. The participants were not allowed to use any antioxidant supplements during the study. All participants received comprehensive oral and written information about the study, but the group allocation was not revealed to them.

### Blood and urine sampling

Blood samples were collected before, during and following altitude camp from a peripheral vein into two EDTA treated tubes by experienced nurses/technicians and centrifuged within 10 minutes at 1500 g after the blood draw. When sampled at sea level, double aliquots of plasma were directly frozen on dry ice and stored at -80° C until analysis. At altitude, the plasma samples were frozen at -20° C for 4–7 days before packaging on dry ice and shipment to University of Oslo where they were stored at -80° C until analysis.

Urine samples were collected before, during and after altitude camp following an overnight fast from the first void after 04:00. Urine samples (1.5mL) were directly frozen on dry ice and stored at -80° C until analysis. Urine samples collected at altitude were frozen at -20° C for 4–7 days before they were shipped on dry ice to University of Oslo and stored at -80° C until analysis.

### Exercise stress-tests

For the assessments of antioxidant capacity and inflammation at rest, and in response to maximal physical exertion stress-test, blood samples were collected after an overnight fast and 10–15 minutes following a VO_2max_ ramp test (for all but swimmers, n = 16) or 100 m all-out swimming (for swimmers, n = 10). The stress-tests were conducted before and after the altitude training camp (Fig 1). All participants were familiar with the exercise tests and followed the exact same standardized, individualized warm-up preceding pre- and post-altitude stress-test (~30 min). In the VO_2max_ ramp test the running speed (10.5% treadmill incline) was increased by 1 km h−1 every minute for the first three minutes, followed by a stepwise increase by 0.5 km h−1 each minute until volitional exhaustion (typical total duration: 5 min 46 s). The swimmers swam 100 m using their favored stroke with maximal effort imitating a standard competition setting (short course pool, 25m). The stress-tests were scheduled at the same time of day pre-and post-altitude for each participant (between 8:30–10 am). Personnel, who analyzed blood and urine samples and conducted the stress-tests were blinded to the group allocation. Detailed information about the VO_2max_ ramp test and 100 m swimming performance and the reason for selecting those as performance/stress tests can be found in our previous article [30]. The dietary intake was recorded 24 hours prior to the stress-tests before altitude, and the participants were asked to replicate the dietary intake prior to testing post altitude.

### Outcome variables

The results on the primary outcome variables have been reported previously [30]. The secondary outcomes of the research project examined in the current study are described below.

#### Ferric reducing ability of plasma (FRAP)

Plasma FRAP is a global indicator of antioxidant capacity. In the plasma, FRAP is primarily sensitive to uric acid, ascorbic acid, and α-tocopherol. Uric acid contributes to 60% of the total FRAP measurements while its role as an endogenous antioxidant is inconclusive [32]. Therefore, FRAP was measured in plasma after removal of uric acid. In the literature this method is referred to as “modified-FRAP analysis” [33]. For preparation of plasma extracts for modified FRAP, 10 μL uricase (0.1 units/10 μL) in Triz buffer (pH 8.5, 400 mmol/L) were added to 25 μL plasma. After incubation for 6 min at room temperature, 80 μL ethanol were added to precipitate proteins. Samples were placed at 4°C for 10 min before centrifugation at 13000 g at 4°C for 10 minutes. The uric acid- and protein free- supernatant were used for modified FRAP analysis. FRAP values were obtained by measuring the reduction of a ferric tripyridyltriazine complex to Ferrous (II) (blue) by absorbance at 593nm 5’ after 5’minutes of incubation as described by Benzie *et al*. [32].

#### Cytokines

Interferon (INF)-γ, tumour necrosis factor (TNF)-α, interleukin (IL)-1α, IL-1β, IL-1RA, IL-2, IL-5, IL-6, IL-7, IL-8, IL-10, IL12p70, IL13, IL17 and monocyte chemoattractant protein (MCP)-1 were measured in plasma by a sandwich immunoassay-based protein array system (Milliplex Human Cytokine/chemokine Magnetic Bead panel assay, CAT HCYTOMAG-60K, USA). Cytokine detection was performed according to the manufacturer’s instructions. Detection was performed with the use of the MAGPIX system, (www.luminexcorp.com) and the xPONENT software was used to process the data. All samples were run in single wells, except the standard curve points, which were run in duplicates. Three kits with identical lot numbers were used for the analyses. In order to avoid eventual batch effects, all time-point samples from each person were analysed using the same kit and stratified based on sports and intervention group. Intra- and inter-assay CVs reported by the manufacturer are 2–13% and 5–19%, respectively [34].

#### Micro-CRP

C-reactive protein was measured with a Hitachi 917 Automated Biochemistry Analyzer. Analytical CV is 1.8% in Fürst laboratory in Oslo, Norway [35].

#### Creatinine

Determination of creatinine in urine was performed by a colorimetric enzymatic principle using a MAXMAT PL II multidisciplinary diagnostic platform and the Creatinine PAP kit, (ERBA Diagnostics, Montpellier, France) as previously described [36].

#### 8-epi-PGF2α (F2-isoprostane)

The determination of the F2-isoprostane 8-epi-PGF2α in urine was performed by liquid chromatography–mass spectrometry as described by Bastani *et al*. [37]. The 8-epi-PGF2α ng/g creatinine ratio was determined by first converting creatinine (mmol/L to g/mL), then dividing 8-epi-PGF2α concentration (ng/mL) with creatinine concentration (g/mL). Creatine adjusted -8-epi-PGF_2α_was used to minimize the potential variation in 8-epi-PGF_2α_ caused by variation in urine volume.

### Statistical analyses

Normally distributed data are presented as mean ± standard deviation (SD), non-normally distributed data as median and range, and categorical data as ranks and percentages. Two samples T-tests or Mann Whitney U Test (for non-normally distributed data) were performed to determine differences in baseline characteristics between the groups.

All values for cytokines, FRAP, 8-epi-PGF_2α_ and micro-CRP were logarithmically transformed. Cytokines that were below the limit of detection were imputed by maximum likelihood estimation which has been described a suitable method for imputation of values below detection limits [38].

The effect of the dietary intervention on the plasma concentrations of cytokines was assessed by linear mixed model regression to account for dependence related to repeated measurements within each subject. The model included cytokines, FRAP or 8-epi-PGF_2α_ as outcome variable, whereas group, timepoint and their interaction term (group × timepoint) were included as fixed effects. Model correction for baseline differences was performed for all cytokines that were significantly different at baseline as indicated in table footnotes. To adjust for random variability among subjects, subject ID was added as a random effect. Because there were no obvious patterns of reliance in the data, and because the Akaike Information Constant was generally lower, an unstructured covariance structure was assumed in the model. Separate models for all outcome variables was created to evaluate the effect of time alone for the total study population and included timepoint as a fixed effect and subject ID as a random effect. These analyses were carried out irrespectively of the interaction analyses. Estimated marginal means and their 95% confidence intervals were extracted from the models and reported for each timepoint per group, along with the nominal p-value for the group × timepoint interaction term. Because some of the models were adjusted for baseline differences, we plotted the relative changes from baseline for illustrative purposes. For micro-CRP, which we measured only on two occasions (pre- and post-altitude), we used ordinary least squares regression to evaluate the difference between groups over time. The grouping variable and baseline micro-CRP were included as covariates in the model. To evaluate the response in plasma cytokine concentrations to exercise stress test (VO_2max_ ramp test or 100 m swimming), we calculated the delta (Δ) (post-test concentration—pre-test concentration). Because the values were logarithmically transformed, the Δ represent the ratio of post-test to pre-test concentrations. All p-values were considered significant at < 0.05. The statistical analyses were carried out using R v.3.0.2 (R for statistical computing, Vienna, Austria), with packages “lme4”, “lmertest” and “emmeans”. Plots were made using the “ggplot2” package.

#### Sample size

This study is part of a larger altitude project [30] where hemoglobin mass (Hb_mass_) was the primary outcome and thus used as the main variable for sample size estimation as previous described [30]. An online calculator was used for the sample size calculation (http://www.powerandsamplesize.com) revealing a need for 7 subjects per group with 1-β = 0.8 and α = 0.05 [39]. The calculation was based on expected increase in Hb_mass_ in response to altitude training by 5.3% [40, 41] and a hypothesized lower adaptive response to altitude training of 1.7% [41] in the intervention group and a standard deviation for change in Hb_mass_ of 2.3% based on previous unpublished data obtained in Norwegian national team athletes.

## Results

### Baseline characteristics

The cohort consisted of 23 males and 8 female Norwegian elite athletes (mean age of 23 years) from five different summer sports: swimming (n = 11), rowing (n = 14), kayaking (n = 4), triathlon (n = 1) and middle-distance running (n = 1). There were no significant differences between the groups in baseline characteristics as presented in Table 1.

### Intervention effects on antioxidant capacity, oxidative stress and biomarkers of inflammation

Estimated marginal means from mixed model analysis and their corresponding confidence intervals for all cytokines, 8-epi-PGF_2α_, FRAP and micro-CRP at each measured timepoint are presented in S1 Table.

**Fig 3:**
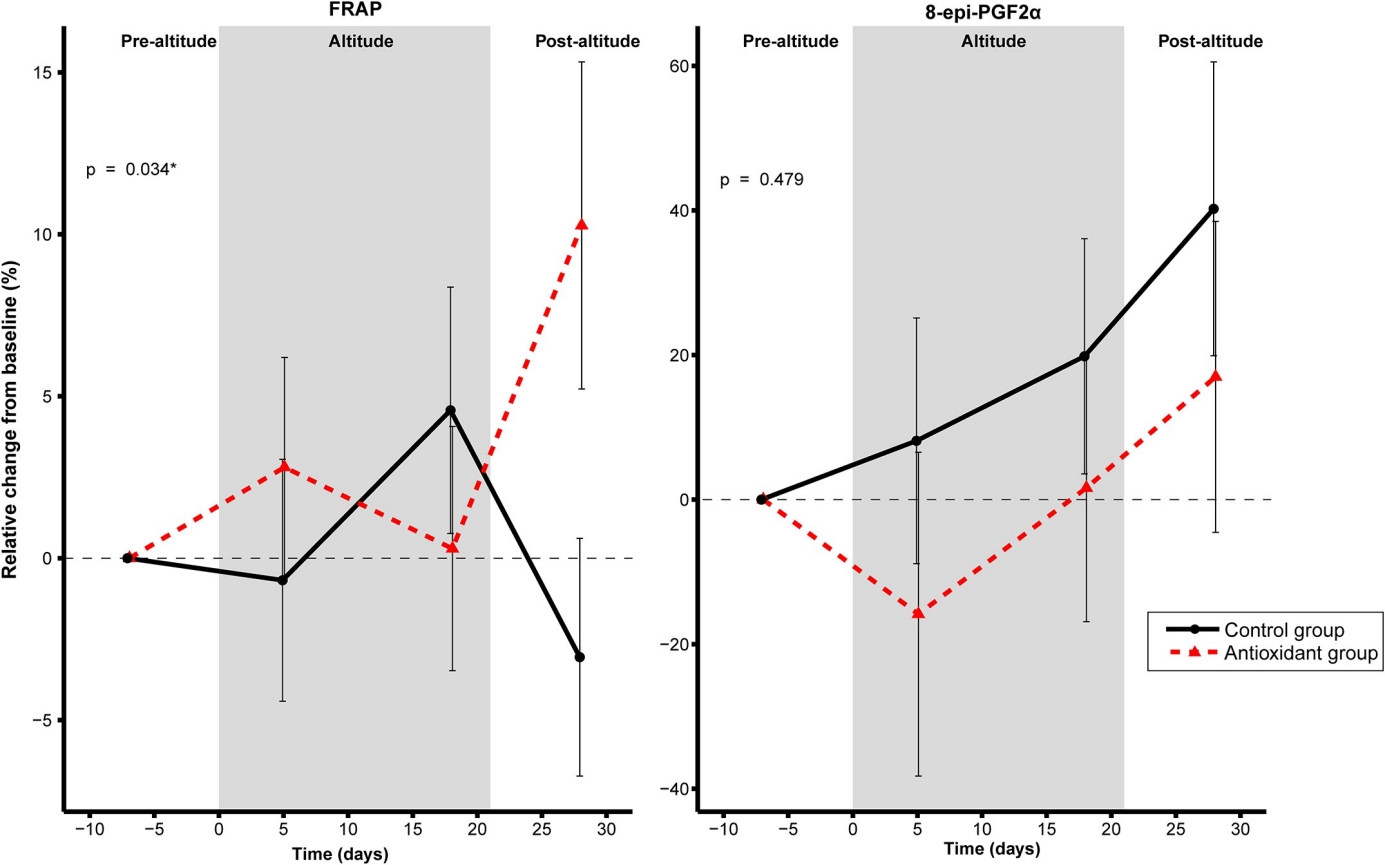
Relative change for FRAP (ferric reducing ability of plasma) and 8-epi-PGF_2α_ in antioxidant and control groups from baseline to day 5 and 18 at altitude, and day 7 post-altitude. Nominal p-value of < 0.05 for the group × timepoint interaction from linear mixed model regression was considered significant. The statistical analyses were carried out using R v.3.0.2 (R for statistical computing, Vienna, Austria), with packages “lme4”, “lmertest” and “emmeans”. Plots were made using the “ggplot2” package.

We found a difference in slopes between antioxidant and control group with a significant group × timepoint interaction for FRAP, where the FRAP increase in the antioxidant group was higher compared to the control group (Fig 3). Post hoc analysis revealed that the increase in FRAP in the intervention group by the post-altitude timepoint contributed to this difference between the groups. 8-epi-PGF_2α_, a marker of oxidative stress, did not change between the groups (p_interaction_ = 0.453) (Fig 3).

**Fig 4:**
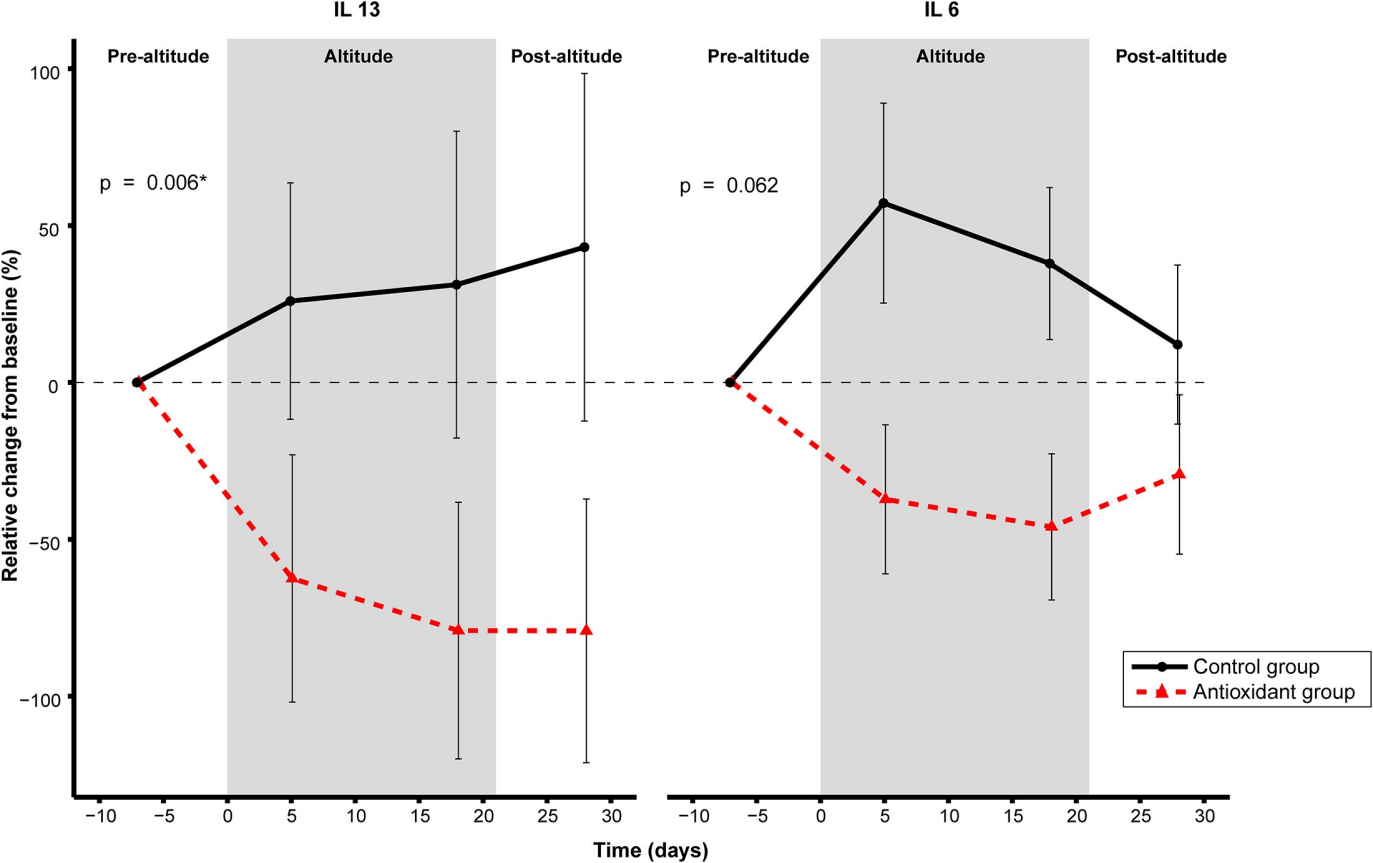
Relative change for IL13 and IL6 in antioxidant and control group during 3-week altitude training camp (2320m). Nominal p-values of < 0.05 for the group × timepoint interaction from linear mixed model regression were considered significant. The statistical analyses were carried out using R v.3.0.2 (R for statistical computing, Vienna, Austria), with packages “lme4”, “lmertest” and “emmeans”. Plots were made using the “ggplot2” package.

We found a difference between the groups with regards to change over time for IL13 where the intervention group decreased, and the control group increased from baseline (group × timepoint, p_interaction_ = 0.006) (Fig 4). A similar trend was found for IL6 (p_interaction_ = 0.062) (Fig 4). The difference in slopes between the groups for IL13 and IL6 became more evident when excluding the post-altitude visit in the model (S2 Table), both showing a significant decrease in the antioxidant group compared to the control (p_interaction_ = 0.023 and 0.006, respectively). There was no difference between the groups with regards to change over time for the rest of the cytokines measured (INF-γ, IL-1α, IL-1β, IL-1RA, IL-2, IL-5, IL-7, IL-8, IL-10, IL12p70). Finally, the change in log transformed plasma concentrations of micro-CRP was significantly different between groups with a larger decrease in micro-CRP in the intervention group compared to controls (β for antioxidant vs. control: -0.62, standard error 0.24, p = 0.02).

### Effects of the altitude training on oxidative stress and biomarkers of systemic inflammation in the total population

We assessed whether there was an effect of time spent at the altitude in the total study population. The regression coefficients and standard errors are presented in S3 Table. We observed a positive effect of time on post-altitude 8-epi-PGF_2α_ (p = 0.033). Also, a significant effect of time was found for IL7 (β: -0.08, p = 0.03) from baseline to post-altitude (S1 Fig).

### Intervention effects on the cytokine response to the exercise stress-tests

Log-transformed cytokine concentrations before and after VO_2max_ ramp test/100 m swimming (n = 26) are presented in S4 Table. We assessed the effects of altitude on the cytokine response to the VO_2max_ ramp test/100 m swimming by comparing the calculated Δ values (post-test concentrations − pre-test concentrations) for all log-transformed cytokines pre- and post-altitude. No significant differences were found between the groups with regards to the change in stress-test response.

### Effects of altitude training on the cytokine response to the exercise stress-tests in the total population

**Fig 5:**
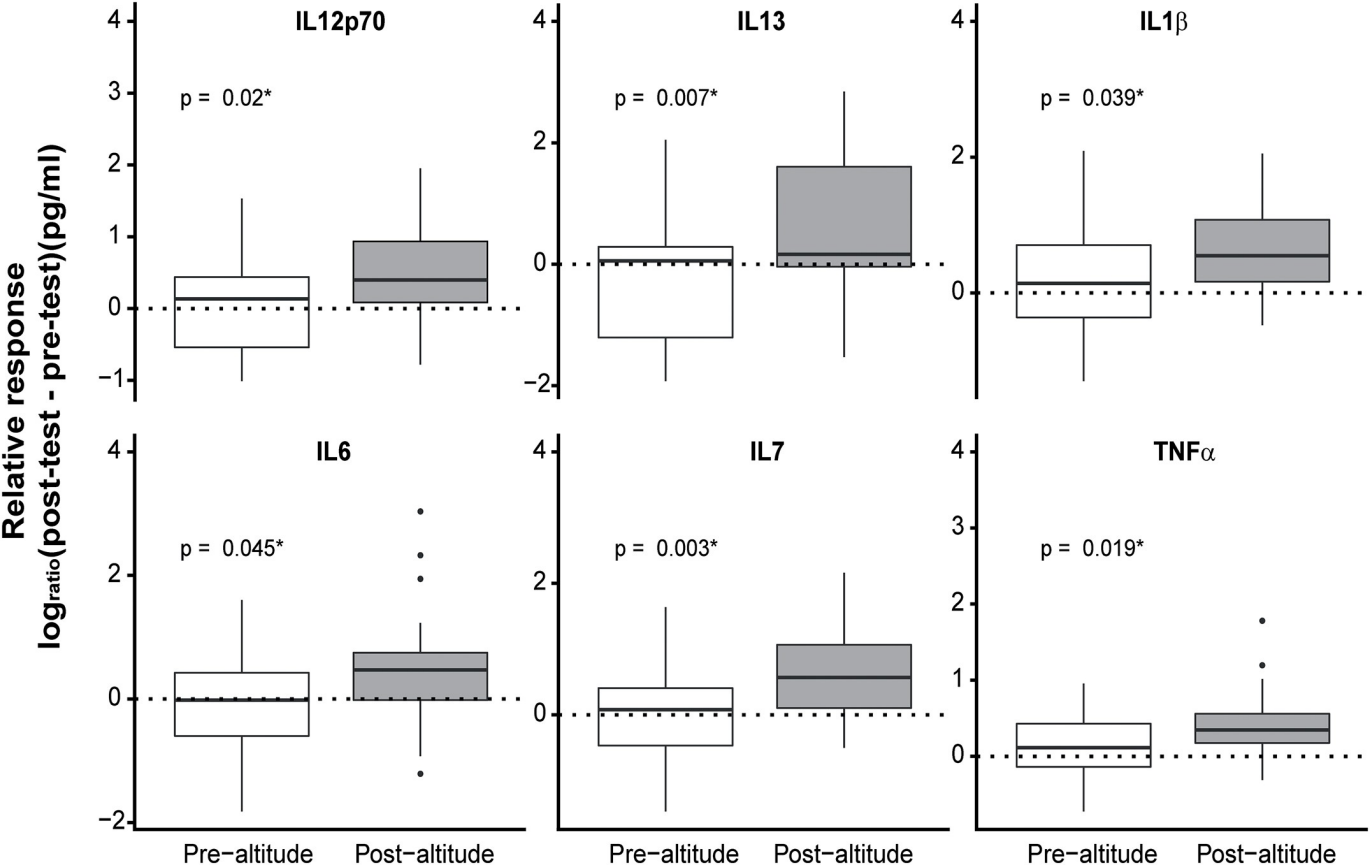
The change in log-transformed plasma cytokine concentrations in response to exercise stress-test (VO_2max_ ramp test/100 m swimming) pre- and post-altitude presented as delta (Δ) (post-test concentration − Pre-test concentration). P-values were calculated using ordinary least squares regression with Δ as the outcome variable and time (post-altitude vs. pre-altitude) as the predictor.

We found significant effects of altitude training in the stress-test response for several cytokines, as shown in Fig 5. Overall, IL1β, IL6, IL7, IL13, IL12p70 and TNFα increased more at the post-altitude VO_2max_ /100 m swimming tests compared to the pre-altitude tests (S4 Table). The pre- and post- delta (Δ) values (post-test concentration − pre-test concentration) are presented with p-values in S5 Table.

## Discussion

This study is the first to examine whether increased intake of antioxidant-rich foods during a 3-week training camp at moderate altitude affects systemic oxidative stress and inflammation at rest and in response to maximal physical exertion in elite endurance athletes.

We show, that it is possible to elevate the plasma antioxidant capacity in elite athletes during training at moderate altitude by increasing daily intake of common antioxidant-rich foods. We also observe that more than doubling the daily antioxidant-rich food intake [30] attenuated the altitude-induced increase in micro-CRP, IL13 and a strong trend for IL6. However, no significant differences were detected between the antioxidant and control group for the remaining measured cytokines or for the altitude-induced increase in 8-epi-PGF_2α_, a biomarker of oxidative stress. The acute inflammatory response to VO_2max_ ramp test/100 m swimming was significantly increased following the 3-week altitude training camp for IL7, IL13, TNFα, IL12p70, IL1β and IL6, although there was no difference in response between the groups.

### Intervention effects on antioxidant capacity (FRAP)

The antioxidant capacity increased more in the antioxidant group compared to the controls. This is in line with previous studies in healthy individuals that show increase in FRAP following acute antioxidant-rich food intake [42–44]. However, the delayed increase in FRAP allows us to speculate that the antioxidant-rich foods lead to a gradual up-regulation of endogenous antioxidant defences, by liberating some of the antioxidant capacity of the molecules detectable by FRAP, rather than exerting an instant free radical scavenging effect. Indeed, it has previously been demonstrated that compounds in fruits and vegetables can modulate gene-expression *in vitro* and *in vivo* [45, 46] and upregulate gene-expression relevant for stress-defences [47]. This may also indicate that a longer exposure of phytochemicals from mixed foods is required for their full biological effect.

Previous studies have reported reduced antioxidant capacity (FRAP) in hypoxia (up to 20%) in well-trained athletes [16, 17, 48]. However, unexpectedly the control group in the current study experienced only a modest decrease in FRAP. Although the total intake of antioxidant-rich foods remained unchanged in the control group, both groups increased their juice intake during the altitude camp [30]. Perhaps, this may have contributed to a lower decrease in FRAP among the controls compared to previous investigations [16, 17, 48].

Another potential explanation could be the upregulation of endogenous antioxidant system (redox-sensitive adaptations) triggered by low-moderate intensity training [49], also described as exercise-induced hormesis [50]. Indeed, recent reviews conclude that hypoxic exercise, especially when performed at low intensities, might concomitantly increase both free radical production and antioxidant capacity [12, 13]. Thus, possibly, rigid control of training intensity of all study participants by their respective national team coaches during the altitude camp may have contributed to a lower reduction in FRAP compared to other trials.

Noteworthy, uric acid in the FRAP analysis was removed because of its role as an endogenous antioxidant is inconclusive [32]. Despite that plasma uric acid has shown to increase following exercise and hypoxia due to purine metabolism in skeletal muscle [13], this xanthine oxidase-driven pathway also includes ROS production and does not appear to be a compensatory mechanism to amplify plasma antioxidant capacity [51]. Given that uric acid contributes to 60% of the total FRAP measurements, FRAP data in the current study is lower compared to literature that has not applied the modified-FRAP assessment [16, 48].

### Intervention effects on oxidative stress (8-epi-PGF_2α_)

We did not find any differences in slopes between the groups for 8-epi-PGF_2α_, a reliable biomarker for lipid peroxidation [52]. This finding is in line with most previous studies that report no effect of antioxidant supplementation on hypoxia-induced oxidative stress [53–55].

We found a 28% increase in 8-epi-PGF_2α_ in the whole population. Previous studies in elite athlete population without antioxidant supplementation have reported both augmented (~60%) [16], or no change [48], in lipid peroxidation (MDA) following 18 days of hypoxia (live high-train low model with simulated altitude). Noteworthy, both studies [16, 48] reported a significant increase in biomarker for oxidative damage to proteins with a concomitant reduction in FRAP. The inconsistency in these findings may be attributed to both type of biomarkers measured as well as the use of various altitude/hypoxic training modalities (terrestrial vs simulated) and differing hypoxic dose [12].

### Intervention effects on cytokines

Our principal new finding revealed that increased antioxidant-rich food intake attenuated the altitude-induced increases in micro-CRP, IL13 and a strong trend for IL6. The altitude-induced increase in IL13 was 54% in the control group, while in the antioxidant-rich food group IL13 reduced by 55%. The response pattern was similar for IL6. Both IL13 and IL6 are important players in the cell mediated immunity. IL13 has both pro- and anti-inflammatory properties [56]. Abnormal expression of IL13 is found in many autoimmune diseases with an inflammatory response [56], while IL6 is released mainly by white blood cells and activates the synthesis of acute phase proteins, like CRP [57]. IL6 is also acutely released by muscle fibers in response to exercise and in response to oxidative stress [58] and is involved in the adaptive response to training. However, chronically elevated IL6 and CRP levels are associated with a wide variety of diseases [59]. The antioxidant intervention in the current study revealed a strong trend for reduction in fasting basal IL6, but it did not alter the acute exercise-induced increase in IL6. This suggests that the intervention had an impact on the basal systemic inflammatory profile of the participants without affecting exercise-induced signaling.

We found a significant altitude-induced increase in IL7 in the whole cohort but no difference was observed between the groups. Given that IL7 is also a myokine, released in response to exercise, we can’t exclude the possibility that some of the cytokine responses could also reflect increase in exercise-induced signaling, rather than a response to altitude alone [60].

The attenuated effects on micro-CRP and IL6 found in the current study are in agreement with the vast body of literature showing anti-inflammatory effects of plant-based foods in in the general population [61, 62], as well as in athletes [25, 63]. Because IL13 is both involved in pro-and anti-inflammatory responses the interpretation of the result is not straight-forward. However, an attenuated IL13 and IL6 response may have particular clinical relevance for respiratory health in endurance athletes, given their central role orchestrating the response to upper respiratory tract infections and implication in asthma [64]. Intriguingly flavonoids have previously been associated with decreased upper respiratory tract infection in healthy individuals [65], although not unambiguously [66].

For the majority of the measured interleukins we did not detect any impact of the antioxidant-rich foods on the altitude-induced cytokine response. The complexity of the cytokine responses, where some have inflammatory and others anti-inflammatory properties, and the various source of their origin (e.g. myocytes, leukocytes) may explain some of this variation [67]. Potentially also the dose and duration of the intervention was insufficient to impact the cytokine response [65]. Finally, the lack of group differences might also be due to the large inter-individual variation in the biomarkers measured. However, the repeated measures design of the current study strengthens the statistical power.

To summarize, the antioxidant intervention in the present study increased antioxidant capacity and attenuated some of the cytokine responses to altitude training but had no detectable impact on the oxidative stress biomarker, 8-epi-PGF_2α_, which increased in both groups. Based on our results, we speculate that the antioxidant intervention only had a marginal effect on free radical scavenging and rather affected other modes of phytochemicals’ actions such as inducing endogenous antioxidant defenses and curtailing the basal systemic inflammatory state of the study participants. The long-term clinical relevance of the altered antioxidant capacity and cytokine response to antioxidant-rich foods in response to altitude training remains to be investigated. Ultimately, minimizing the number of missed workouts due to illness while maximizing the training response are the most relevant measures for endurance athletes and their coaches who utilize altitude training. Thus, future studies should register illness incidence in addition to a spectrum of blood- and muscle-borne inflammatory and oxidative stress indices.

### Limitations

The current study is executed at moderate altitude thus the findings cannot be extrapolated to sojourns at higher altitudes (e.g. mountaineers/climbers > 3000 m). We did not assess activity of antioxidant enzymes, thus, we are not able to provide direct information about the possible alterations in endogenous antioxidant defense. Furthermore, we did not assess oxidative damage to proteins or DNA, limiting conclusions to lipid peroxidation. We did not assess plasma volume changes following the VO_2max_/100 m swimming tests. Thus, there is a possibility for hemoconcentration due to partial dehydration during the stress tests. However, it is unlikely that dehydration would be significant since sweat losses during 30 min warm-up and VO_2max_ramp test/100 m swimming in temperate climate (17°C for VO_2max_ and 27°C for 100 m swim) are expected to be low. Most importantly, however, the interindividual variability in hemoconcentration in the post stress-test sample would have been similar for the antioxidant and control group and should not have affected the group comparisons. Finally, we were not able to obtain biopsies of these athletes due to the close proximity of the Olympic Games, thus information about the cellular impact of antioxidant-rich foods locally in skeletal muscle is lacking.

## Conclusion and perspectives

The present study is the first to examine the impact of increased intake of antioxidant-rich foods on the oxidative and inflammatory response to altitude training (2320 m) in elite endurance athletes. We observed that more than doubling the daily antioxidant intake from natural food sources (e.g. fruit-berry-vegetable smoothies, nuts, dark chocolate and dried fruits/berries) in elite athletes increased the antioxidant capacity but did not affect oxidative stress as measured by 8-epi-PGF_2α_. Also, increased antioxidant-rich food intake attenuated the altitude-induced increases in systematic inflammatory biomarkers (micro-CRP, IL13, IL6), although it did not affect the altitude-induced inflammatory response to exercise stress-test. In addition, since the groups had similar beneficial increases in hemoglobin mass in response to the altitude training as previously reported [30], we suggest that increasing intake of natural antioxidant-rich foods is a sensible addition to elite athlete’s dietary routines while training at moderate altitude.

## Supporting information

S1 Table Estimated mean (95% CI) concentrations of all cytokines, 8-epi-PGF_2α_, FRAP and micro-CRP pre-, during, and post-altitude. (DOCX)

S2 Table Estimated mean (95% CI) concentrations of cytokines, FRAP and 8-epi-PGF_2α_ pre-altitude, day 5 and 18 at altitude. (DOCX)

S3 Table Effect of time on plasma cytokine, 8-epi-PGF_2α_ and FRAP concentrations for the whole population. (DOCX)

S4 Table Cytokine and FRAP concentrations pre and post VO_2max_/100m swimming tests before and after altitude. (DOCX)

S5 Table Response to VO_2max_/100m swimming tests pre- and post-altitude in the total study population. (DOCX)

S1 Fig Relative change from baseline for IL7 for the whole population during the 3-week altitude training camp (2320m). (PDF)

S1 Text Project description approved by the Norwegian Regional Ethics Committee. (PDF)

S2 Text Consort checklist. (PDF)

S3 Text Project description approved by the Norwegian Regional Ethics Committee (Norwegian language version). (DOCX)
