# Supplementary material for: Effects of antioxidant-rich foods on altitude-induced oxidative stress and inflammation in elite endurance athletes: A randomized controlled trial
Source: PLoS One. 2019 Jun 13;14(6):e0217895. doi: 10.1371/journal.pone.0217895 (PMC6563980; doi:10.1371/journal.pone.0217895)
Supplement: S1 Table — (DOCX) [file pone.0217895.s001.docx]

| **S1 Table.**  Estimated mean^1^ (95% CI) concentrations of all cytokines, 8-epi-PGF_2α_, FRAP and micro-CRP pre-, during, and post-altitude. | | | | | | | | | | | |
| --- | --- | --- | --- | --- | --- | --- | --- | --- | --- | --- | --- |
| **Parameter** | **Group** |  | **Pre-altitude** |  | **Altitude_day5_** |  | **Altitude_day18_** |  | **Post-altitude** |  | ***p*_interaction_** |
| IFNγ^2^ (pg/ml) | Control |  | 14.1 (8.87, 22.3) |  | 19.1 (12.1, 30.3) |  | 16.7 (10.5, 26.5) |  | 13.6 (8.57, 21.5) |  | 0.300 |
|  | Antioxidant |  | 17.2 (11.4, 26.0) |  | 21.0 (13.8, 31.8) |  | 20.0 (13.2, 30.3) |  | 20.4 (13.5, 30.9) |  |  |
| IL10^2^ (pg/ml) | Control |  | 16.8 (9.48, 29.7) |  | 16.8 (9.51, 29.7) |  | 18.3 (10.4, 32.4) |  | 13.6 (7.70, 24.1) |  | 0.895 |
|  | Antioxidant |  | 19.2 (11.5, 32.0) |  | 17.4 (10.4, 29) |  | 16.0 (9.57, 26.7) |  | 16.5 (9.87, 27.6) |  |  |
| IL12p70^2^ (pg/ml) | Control |  | 13.0 (7.44, 22.8) |  | 20.0 (11.4, 35.0) |  | 13.4 (7.64, 23.4) |  | 9.85 (5.62, 17.3) |  | 0.400 |
|  | Antioxidant |  | 17.5 (10.5, 29.0) |  | 17.8 (10.7, 29.5) |  | 15.9 (9.61, 26.4) |  | 17.6 (10.6, 29.2) |  |  |
| IL13 (pg/ml) | Control |  | 6.31 (0.92, 43.4) |  | 8.18 (1.19, 56.3) |  | 8.62 (1.25, 59.3) |  | 9.72 (1.41, 66.8) |  | 0.006 |
|  | Antioxidant |  | 8.13 (1.43, 46.2) |  | 4.35 (0.77, 24.7) |  | 3.69 (0.65, 21.0) |  | 3.68 (0.65, 20.9) |  |  |
| IL17 (pg/ml) | Control |  | 6.24 (4.17, 9.33) |  | 9.16 (6.12, 13.7) |  | 8.86 (5.92, 13.3) |  | 5.38 (3.59, 8.05) |  | 0.130 |
|  | Antioxidant |  | 6.53 (4.54, 9.39) |  | 10.6 (7.37, 15.2) |  | 10.3 (7.17, 14.8) |  | 8.09 (5.62, 11.6) |  |  |
| IL1RA (pg/ml) | Control |  | 34.3 (14.6, 80.9) |  | 75.3 (31.9, 177) |  | 73.3 (31.1, 173) |  | 29.0 (12.3, 68.5) |  | 0.571 |
|  | Antioxidant |  | 30.2 (13.9, 65.5) |  | 65.0 (30.0, 141) |  | 63.6 (29.3, 138) |  | 38.1 (17.6, 82.6) |  |  |
| IL1α^2^ (pg/ml) | Control |  | 51.4 (24.5, 108) |  | 68 (32.4, 143) |  | 78.6 (37.5, 165) |  | 54.7 (26.1, 115) |  | 0.944 |
|  | Antioxidant |  | 31.3 (16.1, 60.9) |  | 46.9 (24.1, 91.3) |  | 40.9 (21.0, 79.7) |  | 45.2 (23.2, 88.0) |  |  |
| IL1β (pg/ml) | Control |  | 6.32 (4.24, 9.42) |  | 11.2 (7.53, 16.7) |  | 8.32 (5.58, 12.4) |  | 5.38 (3.61, 8.03) |  | 0.425 |
|  | Antioxidant |  | 6.09 (4.25, 8.72) |  | 10.1 (7.05, 14.5) |  | 8.88 (6.20, 12.7) |  | 6.90 (4.82, 9.89) |  |  |
| IL2 (pg/ml) | Control |  | 5.27 (3.41, 8.14) |  | 6.68 (4.32, 10.3) |  | 5.58 (3.62, 8.62) |  | 4.88 (3.16, 7.54) |  | 0.752 |
|  | Antioxidant |  | 4.92 (3.33, 7.28) |  | 5.68 (3.84, 8.40) |  | 5.24 (3.54, 7.75) |  | 5.27 (3.56, 7.80) |  |  |
| IL5 (pg/ml) | Control |  | 3.68 (1.69, 8.05) |  | 4.77 (2.18, 10.4) |  | 4.64 (2.12, 10.1) |  | 3.23 (1.48, 7.06) |  | 0.643 |
|  | Antioxidant |  | 3.48 (1.72, 7.04) |  | 4.16 (2.06, 8.40) |  | 4.14 (2.05, 8.37) |  | 3.73 (1.84, 7.53) |  |  |
| IL6 (pg/ml) | Control |  | 3.96 (1.76, 8.95) |  | 7.02 (3.11, 15.9) |  | 5.79 (2.56, 13.1) |  | 4.47 (1.98, 10.1) |  | 0.062 |
|  | Antioxidant |  | 5.30 (2.54, 11.0) |  | 3.65 (1.75, 7.61) |  | 3.34 (1.60, 6.97) |  | 3.95 (1.90, 8.23) |  |  |
| IL7 (pg/ml) | Control |  | 8.36 (5.78, 12.1) |  | 10.3 (7.09, 14.8) |  | 9.21 (6.37, 13.3) |  | 5.17 (3.57, 7.48) |  | 0.197 |
|  | Antioxidant |  | 8.07 (5.79, 11.3) |  | 10.3 (7.4, 14.4) |  | 10.0 (7.20, 14.0) |  | 7.05 (5.06, 9.84) |  |  |
| IL8 (pg/ml) | Control |  | 5.76 (2.87, 11.6) |  | 9.68 (4.82, 19.4) |  | 11.7 (5.81, 23.4) |  | 5.84 (2.91, 11.7) |  | 0.389 |
|  | Antioxidant |  | 4.29 (2.29, 8.04) |  | 5.94 (3.17, 11.1) |  | 5.83 (3.11, 10.9) |  | 4.44 (2.37, 8.32) |  |  |
| MCP1 (pg/ml) | Control |  | 219 (192, 251) |  | 248 (217, 284) |  | 266 (233, 305) |  | 199 (174, 228) |  | 0.579 |
|  | Antioxidant |  | 219 (194, 247) |  | 251 (222, 283) |  | 269 (238, 304) |  | 212 (187, 239) |  |  |
| TNFα (pg/ml) | Control |  | 10.4 (7.84, 13.9) |  | 14.2 (10.6, 18.8) |  | 14.2 (10.7, 18.9) |  | 8.64 (6.5, 11.5) |  | 0.492 |
|  | Antioxidant |  | 10.4 (8.06, 13.5) |  | 13.5 (10.5, 17.5) |  | 13.9 (10.7, 18) |  | 10.5 (8.12, 13.6) |  |  |
| 8-epi-PGF_2α_ | Control |  | 758 (556, 1030 |  | 822 (603, 1120 |  | 924 (678, 1260) |  | 1130 (832, 1550) |  | 0.479 |
| (ng/g creatinine) | Antioxidant |  | 978 (725, 1320) |  | 835 (619, 1130) |  | 994 (737, 1340) |  | 1160 (860, 1560) |  |  |
| FRAP (µmol/L) | Control |  | 39.2 (34.8, 44.2) |  | 39.2 (34.8, 44.1) |  | 40.3 (35.8, 45.4) |  | 37.9 (33.6, 42.8) |  | 0.034 |
|  | Antioxidant |  | 37 (33.0, 41.4) |  | 38 (33.9, 42.6) |  | 37.3 (33.3, 41.8) |  | 40.6 (36.2, 45.5) |  |  |
| Micro-CRP^2, 3^ | Control |  | 0.91 (3.35) |  | - |  | - |  | 0.57 (1.97) |  | 0.020 |
| (mg/L) | Antioxidant |  | 0.71 (2.41) |  | - |  | - |  | 0.31 (1.98) |  |  |
| ^1^Estimated marginal means and their confidence intervals were calculated using linear mixed regression model including cytokines as the outcome and group, visit and group*visit as the exposure variables. Abbreviations: IFNγ(Interferon gamma), IL (interleukin), MCP (monocyte chemoattractant protein), TNFα (tumor necrosis factor alpha), FRAP (ferric reducing ability of plasma), CRP (C-reactive protein), PGF [Prostaglandin (PG) F2-like compounds].  ^2^Cytokine concentrations significantly different at baseline. Models including these cytokines are corrected for baseline differences.  ^3^Micro-CRP is presented as geometric means (geometric standard deviations). The p-value was obtained from a linear regression model. | | | | | | | | | | | |
