# Supplementary material for: Effects of antioxidant-rich foods on altitude-induced oxidative stress and inflammation in elite endurance athletes: A randomized controlled trial
Source: PLoS One. 2019 Jun 13;14(6):e0217895. doi: 10.1371/journal.pone.0217895 (PMC6563980; doi:10.1371/journal.pone.0217895)
Supplement: S3 Table — (DOCX) [file pone.0217895.s003.docx]

**S3 Table.**

Effect of time on plasma cytokine, 8-epi-PGF_2α_ and FRAP concentrations for the whole population.

| **Parameter** |  | **β_time_** |  | **SE** |  | ***p*** |
| --- | --- | --- | --- | --- | --- | --- |
| IFNγ |  | 0.00 |  | 0.03 |  | 0.996 |
| IL10 |  | -0.02 |  | 0.04 |  | 0.707 |
| IL12p70 |  | -0.04 |  | 0.04 |  | 0.377 |
| IL13 |  | -0.04 |  | 0.06 |  | 0.482 |
| IL17 |  | -0.01 |  | 0.04 |  | 0.715 |
| IL1 |  | 0.00 |  | 0.07 |  | 0.995 |
| IL1α |  | 0.01 |  | 0.05 |  | 0.886 |
| IL1β |  | -0.03 |  | 0.04 |  | 0.400 |
| IL2 |  | -0.01 |  | 0.03 |  | 0.664 |
| IL5 |  | -0.01 |  | 0.03 |  | 0.725 |
| IL6 |  | -0.03 |  | 0.05 |  | 0.491 |
| IL7 |  | -0.08 |  | 0.04 |  | 0.032 |
| IL8 |  | 0.00 |  | 0.04 |  | 0.937 |
| MCP1 |  | -0.02 |  | 0.01 |  | 0.249 |
| TNFα |  | -0.03 |  | 0.03 |  | 0.340 |
| 8-epi-PGF_2α_ |  | 0.10 |  | 0.05 |  | 0.033 |
| FRAP |  | 0.01 |  | 0.01 |  | 0.250 |
| Beta-coefficients and their standard errors for the effect of time in altitude on plasma interleukin concentrations. Abbreviations: IFNγ (Interferon gamma), IL (interleukin), MCP (monocyte chemoattractant protein), TNFα (tumor necrosis factor alpha), FRAP (ferric reducing ability of plasma), SE (standard error), PGF [Prostaglandin (PG) F2-like compounds]. | | | | | | |
