# Supplementary material for: Effects of antioxidant-rich foods on altitude-induced oxidative stress and inflammation in elite endurance athletes: A randomized controlled trial
Source: PLoS One. 2019 Jun 13;14(6):e0217895. doi: 10.1371/journal.pone.0217895 (PMC6563980; doi:10.1371/journal.pone.0217895)
Supplement: S4 Table — (DOCX) [file pone.0217895.s004.docx]

|  | | | | | | | | |  |
| --- | --- | --- | --- | --- | --- | --- | --- | --- | --- |
|  |  | **Pre-altitude** | |  | **Post-altitude** | |  | |  |
| **Parameter** |  | **Pre-test** | **Post-test** |  | **Pre-test** | **Post-test** |  | ***p*_change_** |  |
| IFNγ (pg/ml) |  | 15.7 (11.5, 21.4) | 17.2 (12.5, 23.7) |  | 17.0 (12.4, 23.3) | 24.5 (17.8, 33.8) |  | 0.058 |  |
| IL10 (pg/ml) |  | 18.1 (12.1, 26.9) | 20.4 (13.6, 30.6) |  | 15.1 (10.4, 22.1) | 24.2 (16.5, 35.6) |  | 0.072 |  |
| IL12p70 (pg/ml) |  | 15.3 (10.7, 22.0) | 16.3 (11.2, 23.6) |  | 13.6 (9.23, 20.0) | 22.8 (15.4, 33.8) |  | 0.020 |  |
| IL13 (pg/ml) |  | 7.26 (2.14, 24.6) | 5.89 (1.73, 20.1) |  | 5.69 (1.73, 18.7) | 10.7 (3.24, 35.5) |  | 0.007 |  |
| IL17 (pg/ml) |  | 6.40 (4.77, 8.58) | 7.57 (5.58, 10.3) |  | 6.73 (5.06, 8.96) | 11.2 (8.34, 15.0) |  | 0.098 |  |
| IL1RA (pg/ml) |  | 32.0 (17.8, 57.6) | 48.4 (26.5, 88.4) |  | 33.7 (19.1, 59.6) | 85.8 (47.7, 154) |  | 0.107 |  |
| IL1α (pg/ml) |  | 39.1 (22.5, 68.0) | 41.1 (23.4, 72.3) |  | 49.2 (30.1, 80.4) | 71.8 (43.5, 119) |  | 0.183 |  |
| IL1β (pg/ml) |  | 6.19 (4.55, 8.42) | 7.27 (5.28, 10.0) |  | 6.18 (4.69, 8.14) | 11.4 (8.59, 15.2) |  | 0.039 |  |
| IL2 (pg/ml) |  | 5.07 (3.82, 6.74) | 5.4 (4.03, 7.25) |  | 5.09 (3.82, 6.79) | 7.27 (5.42, 9.75) |  | 0.079 |  |
| IL5 (pg/ml) |  | 3.57 (2.15, 5.93) | 3.78 (2.26, 6.31) |  | 3.50 (2.01, 6.09) | 4.80 (2.73, 8.42) |  | 0.177 |  |
| IL6 (pg/ml) |  | 4.65 (2.77, 7.80) | 4.62 (2.73, 7.83) |  | 4.18 (2.54, 6.85) | 6.89 (4.15, 11.4) |  | 0.045 |  |
| IL7 (pg/ml) |  | 8.20 (6.23, 10.8) | 8.41 (6.33, 11.2) |  | 6.14 (4.64, 8.11) | 11.4 (8.57, 15.3) |  | 0.003 |  |
| IL8 (pg/ml) |  | 4.89 (3.06, 7.82) | 6.22 (3.87, 9.99) |  | 5.02 (3.20, 7.87) | 8.22 (5.22, 12.9) |  | 0.090 |  |
| MCP1 (pg/ml) |  | 219 (202, 237) | 230 (211, 249) |  | 206 (188, 226) | 242 (220, 266) |  | 0.051 |  |
| TNFα (pg/ml) |  | 10.4 (8.47, 12.8) | 11.9 (9.58, 14.7) |  | 9.62 (7.66, 12.1) | 14.6 (11.5, 18.4) |  | 0.019 |  |
| FRAP (µmol/L) |  | 38.0 (34.9, 41.4) | 59.5 (54.5, 65.0) |  | 40.1 (36.6, 43.9) | 70.3 (63.8, 77.4) |  | 0.164 |  |
| Geometric mean and standard deviation in the total population. P-values were calculated by comparing the post-altitude delta (log-transformed post-test concentration – log-transformed pre-test concentration) to the pre-altitude delta using linear regression. Abbreviations: IFNγ(Interferon gamma), IL (interleukin), MCP (monocyte chemoattractant protein), TNFα (tumor necrosis factor alpha), FRAP (ferric reducing ability of plasma). | | | | | | | | |  |

**S4 Table**

Cytokine and FRAP concentrations pre and post VO_2max_/100m swimming tests before and after altitude.
