# Supplementary material for: Effects of antioxidant-rich foods on altitude-induced oxidative stress and inflammation in elite endurance athletes: A randomized controlled trial
Source: PLoS One. 2019 Jun 13;14(6):e0217895. doi: 10.1371/journal.pone.0217895 (PMC6563980; doi:10.1371/journal.pone.0217895)
Supplement: S5 Table — (DOCX) [file pone.0217895.s005.docx]

**S5 Table.** Response to VO_2max_/100m swimming tests pre- and post-altitude in the total study population.

|  | | | | | | |
| --- | --- | --- | --- | --- | --- | --- |
|  | | | | | | |
| **Parameter** |  | **Pre-altitude Δ** |  | **Post-altitude Δ** |  | ***pΔ_interaction_*** |
| IFNγ(pg/ml) |  | 1.09 (1.81) |  | 1.45 (1.59) |  | 0.058 |
| IL10 (pg/ml) |  | 1.11 (2.09) |  | 1.60 (1.94) |  | 0.072 |
| IL12p70 (pg/ml) |  | 1.05 (1.96) |  | 1.69 (2.07) |  | 0.020 |
| IL13 (pg/ml) |  | 0.81 (2.95) |  | 1.91 (3.03) |  | 0.007 |
| IL17 (pg/ml) |  | 1.19 (2.18) |  | 1.66 (1.88) |  | 0.098 |
| IL1RA (pg/ml) |  | 1.53 (3.04) |  | 2.63 (3.58) |  | 0.107 |
| IL1α (pg/ml) |  | 1.06 (2.46) |  | 1.48 (2.37) |  | 0.183 |
| IL1β (pg/ml) |  | 1.17 (2.35) |  | 1.85 (1.98) |  | 0.039 |
| IL2 (pg/ml) |  | 1.08 (1.90) |  | 1.43 (1.65) |  | 0.079 |
| IL5 (pg/ml) |  | 1.06 (1.86) |  | 1.38 (2.16) |  | 0.177 |
| IL6 (pg/ml) |  | 0.99 (2.34) |  | 1.64 (2.49) |  | 0.045 |
| IL7 (pg/ml) |  | 1.03 (2.01) |  | 1.88 (1.97) |  | 0.003 |
| IL8 (pg/ml) |  | 1.28 (1.77) |  | 1.65 (1.62) |  | 0.090 |
| MCP1 (pg/ml) |  | 1.05 (1.22) |  | 1.18 (1.26) |  | 0.051 |
| TNFα (pg/ml) |  | 1.13 (1.56) |  | 1.52 (1.56) |  | 0.019 |
| FRAP (µmol/L) |  | 1.56 (1.29) |  | 1.73 (1.33) |  | 0.164 |
| Geometric mean and standard deviation in the total population. Values are expressed as log-ratios (ratio of post- VO_2max_/100 m swimming to pre- VO_2max_/100 m swimming). P-values were calculated using a linear regression model with response as outcome and time as predictor. Abbreviations: IFNγ (Interferon gamma), IL (interleukin), MCP (monocyte chemoattractant protein), TNFα (tumor necrosis factor alpha), FRAP (ferric reducing ability of plasma). | | | | | | |
