# Supplementary material for: Effects of antioxidant-rich foods on altitude-induced oxidative stress and inflammation in elite endurance athletes: A randomized controlled trial
Source: PLoS One. 2019 Jun 13;14(6):e0217895. doi: 10.1371/journal.pone.0217895 (PMC6563980; doi:10.1371/journal.pone.0217895)
Supplement: S1 Text — (PDF) [file pone.0217895.s007.pdf]

PROJECT DESCRIPTION - Antioxidant-rich diet and altitude training

The effect of increased intake of antioxidant-rich foods on exercise adaptation, redox balance and inflammation among top athletes during a moderate altitude training session (2300m)

Koivisto A[1], Siv Kjølsrud Bøhn[2], Ingvild Paur[2], Gøran Paulsen[1], Espen Tønnesen[1], Ina Garthe[1], Truls Raastad[3], Rune Blomhoff[2]

[1]Norwegian Olympic Sports Centre, Sognsveien 228, 0806 Oslo, [2]Department of nutrition, Faculty of Medicine, Universitety of Oslo, [3] Dept. of Physical Performance, Norwegian School of Sport Sciences, Oslo, Norway

Summary

Athletes, especially practicipating in endurance sports, are at increased risk of oxidative stress and inflammation-related diseases and injuries. During exercise, and especially during exercise at altitude, the production of reactive oxygen and nitrogen compounds (RONS) and inflammation markers increases. Intake of antioxidant supplements has become a common nutritional strategy among athletes who believe that it is a plausible measure to prevent oxidative stress and oxidative damage. However, a transient increase of RONS is required to activate signal cascades that initiate training adaptation. Several independent studies have shown that antioxidant supplements hamper training adaptation, possibly by interfering with / reducing the signalling cascades initiated by RONS. However, it is not known whether a high intake of antioxidant-rich foods can affect the overproduction of RONS, inflammatory markers and / or exercise adaptation. We therefore want to investigate whether an increased intake of natural antioxidants in the form of fruits, vegetables and berries, in line with the official Norwegian dietary guidelines, can affect antioxidant status, immune defense and training adaptation in connection with altitude training in Norwegian elite athletes who are candidates for the 2016 Olympics Games.

Background

Reactive oxygen and nitrogen compounds (RONS) are continuously formed as a result of natural cell metabolism. The body's antioxidant defense can usually neutralize and disarm these compounds before the cell's lipids, proteins and DNA become damaged, but if there is an imbalance between RONS and the cell's antioxidant capacity, it will cause a state of oxidative stress. Many chronic lifestyle diseases are associated with oxidative stress (Hu 2003), and athletes, especially in endurance sports, are at increased risk of oxidative stress and inflammation-related diseases and injuries (Carlsen, Hem et al. 2011, Metz, Wustrack et al. 2012, Turagam, Velagapudi et al. 2012, Dean, Gettings et al. 2015, Elkington, Gleeson et al. 2015, Sugama, Suzuki et al. 2015). During exercise, production of RONS increases in a dose-dependent manner (Knez, Jenkins et al. 2014). This can result in disrupted cell function and contribute to muscular fatigue and possibly delayed recovery (Powers and Jackson 2008). Antioxidants, a common term for molecules with the ability to protect other molecules from being oxidized, thus have the potential to counteract oxidative stress. Therefore, many athletes believe that it is necessary to

consume large amounts of antioxidants in the form of supplements, to protect themselves from exercise-induced overproduction of RONS. Despite possible negative effects of oxidative stress, a transient increase in RONS is an important signal for exercise adaptation. RONS seems to activate multiple signal molecules, including PGC-1α, which is a chief regulator of mitochondrial biogenesis (Powers, Duarte et al. 2010). Previous studies have shown that large single dose antioxidant supplements can impair the adaptive response to training(Gomez-Cabrera, Domenech et al. 2008, Ristow, Zarse et al. 2009, Paulsen, Cumming et al. 2014). Therefore, it is speculated that antioxidant supplements prevent exercise-induced adaptation by interfering with the signal cascades initiated by RONS.

It is not known whether a high intake of natural antioxidants via ingestion of fruits and vegetables can have similar undesirable effects on training adaptation. A high intake of fruits and vegetables, naturally rich in antioxidants, protects against a variety of diseases associated with oxidative stress. In addition, an antioxidant-poor diet, e.g. low intake of fruits and vegetables, among athletes has been associated with increased levels of systemic inflammatory markers (Watson, Callister et al. 2005, Plunkett, Callister et al. 2010). Since fruits and vegetables are rich in antioxidants, it has been suggested that food-based antioxidants provide additional protection against oxidation in the body. However, since fruits and vegetables contain tens of thousands of different plant chemicals (phytochemicals), it is not possible to conclude that the antioxidants are responsible for the disease protection. Most phytochemicals have antioxidant properties, but they also have other properties that can have different effects in the body. Among other things, other properties of the phytochemicals can be important for optimizing the body's own defense systems (Bøhn, Myhrstad et al. 2010).

At the Norwegian Olympic Sports Centre, athletes are advised not to use high doses of antioxidant supplements. In general, it is recommended to increase the intake of antioxidant-rich foods during periods of high exercise load as a strategy to avoid negative effects of oxidative stress. Norwegian health authorities recommend at least five servings of fruit, berries and vegetables per day (500g) (Helsedirektoratet, 2014). During an altitude training session in June 2015 it was revealed that the intake of fruit and vegetables among elite athletes is far below the Heath Authorities recommendations. Furthermore, it is uncertain whether the recommended "five a day" provides a sufficient intake of antioxidants for endurance athletes during their most demanding training periods.

In order to test whether natural antioxidants from plant foods have an effect on exercise adaptation, we want to perform a clinical controlled study on athletes exposed to extra high oxidative stress levels at moderate altitude (2300 m). Altitude exposure, which reduces oxygen availability (hypoxia), has previously been shown to increase the oxidative stress levels (Pialoux, Mounier et al. 2009, Pialoux, Mounier et al. 2009).

Studies using the "live high - train low" model (training at 1200m and living at 2500-3000m simulated height) among elite athletes have shown increased oxidative stress. At the same time, the antioxidant capacity in serum has been reduced when the duration of altitude exposure has passed 18 days (Pialoux, Mounier et al. 2009, Pialoux, Mounier et al. 2009).

Given that exposure to altitude and increased exercise load will increase oxidative stress levels, it is possible that athletes' redox balance can be changed during altitude training (the "live high-train high" method). Altitude training will also strain the body's immune system. Previous studies have shown that

exercise in hypoxia increases secretion of some cytokines and leads to transient immunosuppression (Mazzeo 2005).

Finally, it is unknown how an antioxidant-rich diet will affect exercise adaptation, antioxidant balance, immune defense and gene expression during altitude training. There is lack of data on the general antioxidant intake during altitude training in elite athletes. There is also a lack of reference values for biomarkers for antioxidant capacity and oxidative stress in elite athletes (Lewis, Howatson et al. 2015).

Aims of the study

The purpose of the study is to investigate whether an antioxidant-rich diet can impact the health-related effects of altitude training in elite athletes by optimizing snack foods in the intervention group for intake of antioxidant-rich foods during a 3-week training camp in moderate altitude (2300 m). Specifically, we want to explore the dietary intake of antioxidants, and oxidative stress and antioxidant capacity before, during and after the altitude training camp. Furthermore, we want to study the effects of an antioxidant-rich diet on redox balance, inflammation status and exercise adaptation during a 3-week altitude training camp. With a special focus on the stress response (the body's own defense systems) we will study how the altitude sojourn affects the gene expression profile and whether the intervention affects these defense systems compared to the controls.

Specific hypotheses

1. Adaptation to altitude measured as increased hemoglobin mass will not be affected by increased intake of antioxidant rich meals.

2. Sports performance will have a more positive development during the altitude training camp following intake of antioxidant-rich foods, because muscles and the circulatory system will better withstand the combination of hypoxia and large exercise volume.

3. Athletes have suboptimal intake of fruits, berries and vegetables (<500g per day) in lowland and at altitude.

4. The altitude exposure combined with large exercise volume will increase oxidative stress and reduce antioxidant capacity. This will result in increased levels of inflammatory biomarkers.

5. Daily intake of antioxidant-rich foods will increase circulating levels of selected antioxidants and limit the increase in oxidative stress and inflammation biomarkers.

6. Altitude training results in changes in the gene expression profile in blood cells, especially genes involved in the stress response. Altitude-induced changes in gene expression profile are different between the groups.

Outcome variables

Effect of altitude training camp: e.g. hemoglobin mass and blood volume, maximum oxygen uptake (VO2max), lactate profile, performance in sport-specific tests.

• Anthropometry: e.g. body weight, lean body mass (LBM), fat mass (FM), bone density (BMD)

• Diet: e.g. energy, macronutrients, micronutrients, number of servings of fruits, vegetables and berries, as well as total antioxidant intake (mmol)

• Compliance: biomarkers for fruit and vegetable intake (e.g. carotenoids)

• Inflammation markers: e.g. IL-1, IL-6, TNFα

• Gene expression: Whole genomic gene expression

• Antioxidant status:

  o Oxidative stress biomarkers: e.g. lipid peroxidation (e.g. 8-isoPGF2α).

  o Antioxidant capacity: e.g. ferric reducing ability of plasma (FRAP)

- Total oxidative stress: e.g. dROM

• Antioxidants in plasma: e.g. carotenoids, flavonoids

• Other data from routine medical screening of athletes (e.g., estradiol, testosterone, crp, cortisol, hb, ferritin, hematocrit, vitamin D, vitamin E, B12, folate, cholesterol, HDL, LDL)

Participants

Inclusion criteria

National team athletes, men and women (18-42 years) from paddling, rowing, cycling, swimming and triathlon who will attend a height gathering for the 2016 Olympics. Current elite athletes in endurance sports will be asked to participate.

Exclusion criteria

- Allergies to the relevant foods where it is not possible to replace foods with similar antioxidant-rich products in consultation with the study participant.
- Use of medications that can affect exercise and performance tests.
- Injuries that may affect exercise and performance tests.

Recruitment

Participants will be recruited among endurance athletes that have the potential to participate in Olympic Games 2016. The athletes will be randomized to an intervention group or control group stratified for sport category and gender.

Power Calculations

Based on a power size of 1.0, with a two-sided significance level of 5% and 80% power, we need 17 athletes in each group. Estimated change in effect size is based on the variation in performance tests in 2000 m rowing on the rowing-ergometer where the variation from day to day is 1.3% (SD) under otherwise equal conditions. A change in performance of 1.3% corresponds to approx. 5 second improvement of 2000 m rowing; an improvement that can separate medal winners in an Olympics. The same relative changes are expected in performance on the other sports represented in the experiment. If we calculate a dropout of 10%, we will need 19 in each group to detect significant differences between the groups. We want to invite all current practitioners to participate (n = 45) because we expect 85% to agree to participate.

Timeline

Participants will be recruited four weeks prior to the altitude training camp, at the end of September 2015. The intervention and altitude training camp in Spain will end on November 11, 2015. All participants will undergo a general medical examination and obtain clinical parameters three weeks ahead of altitude stay. In addition, hemoglobin mass and body composition are assessed one week before and after the altitude camp. Repeated records of diet, anthropometry and performance, as well as blood sampling, will be carried out before, during and after the altitude camp (Figure 1).

Figure 1. Timeline of the study.

UKE -3	UKE -2	UKE -1	UKE 1	UKE 2	UKE 3	UKE 4
Medisinsk us		DXA	HØYDEOPPHOLD (2300m)			DXA
Jernstatus		Blodvolum<br>VO2 max<br>Laktat profil	INTERVENSJON<br>Daglig inntak av antioksidantrike matvarer			Blodvolum<br>VO2 max<br>Laktat profil
		Prestasjonstester	Prestasjonstester		Prestasjonstester	Prestasjonstester
		Blodprøver	Blodprøver		Blodprøver	Blodprøver
		Urinprøve	Urinprøve		Urinprøve	Urinprøve
24 h recall		2 x 24 h recall	24 h recall	24 h recall	24 h recall	

Methods

The intervention

The purpose of the intervention is to optimize the in between-meals foods for athletes during the altitude training camp, specifically regarding the intake of antioxidant-rich foods. The intervention group will receive foods naturally high in antioxidants (including berries, nuts, dark chocolate, vegetables and

fruits) (Carlsen, Halvorsen et al. 2010). The control group will receive foods that are usually consumed as in-between meals during the altitude camp. The calorie content of the snacks provided to the groups will be matched (~1000 kcal), and will be delivered once a day to each individual participant. The participants in both groups are otherwise encouraged to maintain stable body weight (avoid body weight abnormalities > 2%) throughout the entire altitude camp.

The in-between meals in the intervention group (Daily)

The aim is to double the antioxidant intake of the practitioners in the intervention group by offering a mix of antioxidant-rich snacks. The intervention described below is an example. Other common antioxidant-rich foods may also be included.

- 3 pcs fruit / berry / vegetable smoothie selected from the following products (450 kcal):
    - Bama, Turn 250 ml (71 kcal / 100ml): 44% grape juice, 30% banana puree, 17% pomegranate juice and 9% black currant juice
    - Bama, Slide 250 ml (47 kcal / 100ml): 38% apple juice, 20% carrot puree, 20% mango puree 20%, 17% apple puree and 5% passion fruit puree
    - Bama, Spin 250 ml (62 kcal / 100ml): 38% apple juice, 21% red grape juice, 20% banana puree, 8% blueberry puree, 8% black currant juice and 5% apple puree
- 1 dl (40 g) walnuts (270 kcal)
- 1 dl (50g) mixture of dried fruit and berries; cranberries, goji berries, apricots (Rema1000 and Bama) (140 kcal)
- 40g dark chocolate (70%), selection of Freia Premium chocolates in several flavors; plain, pecan and sea salt, orange, almond, hazelnut (240kcal)

The in-between meals in the control group (Daily)

- 220 ml TINE milkshake in several flavors; strawberry and banana or cocoa (160 kcal)
- 330ml TINE YT recovery drink in several flavors; strawberry and banana or cocoa (220 kcal)
- 4 Bixit biscuits (60 g), or other type of sweet biscuits without fillings (290 kcal)
- 1 dl (30g) Ritz salt biscuits / salt bars (120 kcal)
- 50g Freia white chocolate (270 kcal)

Antropometry

Standardized DXA measurements (dual energy x-ray; iDXA; GE Healthcare) is taken before and after exercise camp to measure muscle mass (LBM), fat mass (FM) and bone density (BMD). Body weight with the least possible amount of clothing before breakfast is recorded daily and the practitioners are encouraged to maintain a stable body weight (avoid body weight abnormalities> 2%) throughout the altitude camp.

Dietary recordings

Four 24-hour recalls are conducted in an interview session before the altitude camp and three times during the altitude camp by the same registered dietitian. 24h recalls will be separated by at least 2 days in time. Interview guide for 24h recall follows in a separate attachment.

Blood sampling

Fasting blood samples with venipuncture are collected before and after elevation collection and on two test days during the altitude camp. In addition, weekly blood samples are taken from the fingertip during the height collection. Dried Blood Spot method is used which enables measurements of various biomarkers in a less invasive manner. The laboratory at UiO will analyze the blood samples with regard to biomarkers such as oxidative damage biomarkers, biomarkers of inflammatory status, antioxidant status and gene expression in blood cells. The protocol for blood sampling and handling is included in a separate appendix. Whole blood gene transcription assays, low density gene arrays and / or RT-PCR will be performed on blood cells collected at the various times.

Bloodvolume

Hemoglobin mass, plasma volume and total blood volume will be estimated by the CO-rebreathing method after Burge and Skinner (Burge and Skinner 1995). This procedure will be performed twice before and after the intervention period at altitude.

Physiological tests and performance tests

Participants in this study are all elite athletes with affiliation to the Norwegian Olympic Sports Center. Thus, all the athletes are experienced with the tests described below.

The performance test and maximum oxygen uptake (VO2max) will only be tested at the Norwegian Olympic Sports Center (at sea-level) before and after the intervention period at altitude. The lactate profile test (without oxygen uptake measurements) will also be carried out at altitude: 4-5 days after the participants reach the altitude and 2-3 days before returning home.

Lactate Profile Test

Participants will perform a lactate profile test in a sports-specific ergometer / element (bicycle, rowing and paddle device, or water). This is carried out according to the standard procedure at Norwegian Olympic Sports Center (Stoa, Storen et al. 2010).

After 10 minutes of heating at 60-70% of HF-max, 4 to 6 interval drafts are performed in 5 minutes with increasing intensity. The test ends when the blood lactate value is 1.5 mmol higher than the lactate value on the first two intervals. Between each interval draw there is a 30 second break. Lactate is measured in whole blood taken by fingerstick. The fingertip is washed and dried before each sample is taken. Oxygen uptake (see below) and heart rate are recorded along the way on each interval draw.

VO_{2max}

In the extension of the lactate profile test, a VO2max test is carried out according to the standard procedure at Norwegian Olympic Sports Centre (Tonnessen, Haugen et al. 2015). The test starts at one intensity level under anaerobic threshold, and the intensity is increased every minute. The test is continued until exhaustion, and typically lasts for 4-6 minutes. Swimmers will measure VO2max with uphill running on a treadmill.

Sport -specific test

In a sports-specific ergometer / element, the participants will perform a distance test, where the goal is to produce as high average watt / speed as possible. The duration will vary from about 2 min to 15 min depending on the participant's sport.

- Cycling: 10,000 m on a cycling roller
- Rowing: 2000 m in Concept2 radar
- Paddling: 1000 m Concept2 paddle device
- Swimming: 200 m in water (own swimming species)

Heart rate will be measured continuously while lactate will be measured 1 min after the test.

Training diary records

Before, during and after the altitude camp, the athletes log their training in the Norwegian Olympic Sports Centre's online training diary. The training diary records the duration and intensity of the training, as well as the form of exercise and the form of movement. The training is documented in the same manner as previously described (Tonnessen, Sylta et al. 2014).

Overview of the study protocol visits

Visit 1: The measurements take place at the health department at Norwegian Olympic Sports Centre in Oslo.

- General medical examination
- Blood tests (venous)

- Measurement of hemoglobin mass
- 24 hour recall (see attachment)

Visit 2: The measurements take place at the health department and test lab at Norwegian Olympic Sports Centre in Oslo.

- Fasting DXA measurement (at Norwegian School of Sport Sciences)
- Measurement of hemoglobin mass
- 24h recall
- Lactate Profile
- VO2max

Visit 3: The measurements take place at the health department and test lab at Norwegian Olympic Sports Centre in Oslo.

- Fasting blood samples (venous and fingertip) and urine sample
- Blood tests after breakfast (venous and fingertip)
- Performance Tests
- Blood tests after performance tests (venous and fingertip)

• 24h recall

Visit 4: The measurements take place in Centro de alto rendimento in Sierra Nevada, Spain.

- Fasting blood samples (venous and fingertip) and urine sample
- Blood tests after breakfast (venous and fingertip)
- Lactate Profile
- Blood tests after lactate profile (venous and fingertip)
- 24h recall

Visit 5: The measurements take place in Centro de alto rendimento in Sierra Nevada, Spain.

- Fasting blood samples (venous and fingertip) and urine sample
- Blood tests after breakfast (venous and fingertip)
- Lactate Profile
- Blood tests after lactate profile (venous and fingertip)
- 24h recall

Visit 6: The measurements take place at the health department and test lab at Norwegian Olympic Sports Centre in Oslo.

- Fasting blood samples (venous and fingertip) and urine sample
- Measurement of hemoglobin mass
- Blood tests after breakfast (venous and fingertip)
- Performance Tests
- Blood tests after performance tests (venous and fingertip)

Visit 7: The measurements take place at the health department and test lab at Norwegian Olympic Sports Centre in Oslo

- Fasting DXA measurement
- Measurement of hemoglobin mass
- 24h recall
- Lactate Profile
- VO2max

References

Burge, C. M. and S. L. Skinner (1995). "Determination of hemoglobin mass and blood volume with CO: evaluation and application of a method." J Appl Physiol (1985) **79**(2): 623-631.

Bøhn, S. K., M. C. Myhrstad, M. Thoresen, M. Holden, A. Karlsen, S. H. Tunheim, I. Erlund, M. Svendsen, I. Seljeflot, J. O. Moskaug, A. K. Duttaroy, P. Laake, H. Arnesen, S. Tonstad, A. Collins, C. A. Drevon and R. Blomhoff (2010). "Blood cell gene expression associated with cellular stress defense is modulated by antioxidant-rich food in a randomised controlled clinical trial of male smokers." BMC Med **8**: 54.

Carlsen, K. H., E. Hem and T. Stensrud (2011). "Asthma in adolescent athletes." Br J Sports Med **45**(16): 1266-1271.

Carlsen, M. H., B. L. Halvorsen, K. Holte, S. K. Bohn, S. Dragland, L. Sampson, C. Willey, H. Senoo, Y. Umezono, C. Sanada, I. Barikmo, N. Berhe, W. C. Willett, K. M. Phillips, D. R. Jacobs, Jr. and R. Blomhoff (2010). "The total antioxidant content of more than 3100 foods, beverages, spices, herbs and supplements used worldwide." Nutr J **9**: 3.

Dean, B. J., P. Gettings, S. G. Dakin and A. J. Carr (2015). "Are inflammatory cells increased in painful human tendinopathy? A systematic review." Br J Sports Med.

Elkington, L. J., M. Gleeson, D. B. Pyne, R. Callister and L. G. Wood (2015). Inflammation and Immune Function: Can Antioxidants Help the Endurance Athlete? Antioxidants in Sport Nutrition. M. Lamprecht. Boca Raton FL, 2015 by Taylor & Francis Group, LLC.

Gomez-Cabrera, M. C., E. Domenech, M. Romagnoli, A. Arduini, C. Borras, F. V. Pallardo, J. Sastre and J. Vina (2008). "Oral administration of vitamin C decreases muscle mitochondrial biogenesis and hampers training-induced adaptations in endurance performance." Am J Clin Nutr **87**(1): 142-149.

Hu, F. B. (2003). "Plant-based foods and prevention of cardiovascular disease: an overview." Am J Clin Nutr **78**(3 Suppl): 544S-551S.

Knez, W. L., D. G. Jenkins and J. S. Coombes (2014). "The effect of an increased training volume on oxidative stress." Int J Sports Med **35**(1): 8-13.

Lewis, N. A., G. Howatson, K. Morton, J. Hill and C. R. Pedlar (2015). "Alterations in redox homeostasis in the elite endurance athlete." Sports Med **45**(3): 379-409.

Mazzeo, R. S. (2005). "Altitude, exercise and immune function." Exerc Immunol Rev **11**: 6-16.

Metz, L. N., R. Wustrack, A. F. Lovell and A. J. Sawyer (2012). "Infectious, inflammatory, and metabolic diseases affecting the athlete's spine." Clin Sports Med **31**(3): 535-567.

Paulsen, G., K. T. Cumming, G. Holden, J. Hallen, B. R. Ronnestad, O. Sveen, A. Skaug, I. Paur, N. E. Bastani, H. N. Ostgaard, C. Buer, M. Midttun, F. Freuchen, H. Wiig, E. T. Ulseth, I. Garthe, R. Blomhoff, H. B. Benestad and T. Raastad (2014). "Vitamin C and E supplementation hampers cellular adaptation to endurance training in humans: a double-blind, randomised, controlled trial." J Physiol **592**(Pt 8): 1887-1901.

Pialoux, V., R. Mounier, J. V. Brugniaux, E. Rock, A. Mazur, J. P. Richalet, P. Robach, J. Coudert and N. Fellmann (2009). "Thirteen days of "live high-train low" does not affect prooxidant/antioxidant balance in elite swimmers." Eur J Appl Physiol **106**(4): 517-524.

Pialoux, V., R. Mounier, E. Rock, A. Mazur, L. Schmitt, J. P. Richalet, P. Robach, J. Brugniaux, J. Coudert and N. Fellmann (2009). "Effects of the 'live high-train low' method on prooxidant/antioxidant balance on elite athletes." Eur J Clin Nutr **63**(6): 756-762.

Plunkett, B. A., R. Callister, T. A. Watson and M. L. Garg (2010). "Dietary antioxidant restriction affects the inflammatory response in athletes." Br J Nutr **103**(8): 1179-1184.

Powers, S. K., J. Duarte, A. N. Kavazis and E. E. Talbert (2010). "Reactive oxygen species are signalling molecules for skeletal muscle adaptation." Exp Physiol **95**(1): 1-9.

Powers, S. K. and M. J. Jackson (2008). "Exercise-induced oxidative stress: cellular mechanisms and impact on muscle force production." Physiol Rev **88**(4): 1243-1276.

Ristow, M., K. Zarse, A. Oberbach, N. Kloting, M. Birringer, M. Kiehntopf, M. Stumvoll, C. R. Kahn and M. Bluher (2009). "Antioxidants prevent health-promoting effects of physical exercise in humans." Proc Natl Acad Sci U S A **106**(21): 8665-8670.

Stoa, E. M., O. Storen, E. Enoksen and F. Ingjer (2010). "Percent utilization of VO2 max at 5-km competition velocity does not determine time performance at 5 km among elite distance runners." J Strength Cond Res **24**(5): 1340-1345.

Sugama, K., K. Suzuki, K. Yoshitani, K. Shiraishi, S. Miura, H. Yoshioka, Y. Mori and T. Kometani (2015). "Changes of thioredoxin, oxidative stress markers, inflammation and muscle/renal damage following intensive endurance exercise." Exerc Immunol Rev **21**: 130-142.

Tonnessen, E., T. A. Haugen, E. Hem, S. Leirstein and S. Seiler (2015). "Maximal Aerobic Capacity in the Winter Olympic Endurance Disciplines: Olympic Medal Benchmarks for the Time Period 1990-2013." Int J Sports Physiol Perform.

Tonnessen, E., O. Sylta, T. A. Haugen, E. Hem, I. S. Svendsen and S. Seiler (2014). "The road to gold: training and peaking characteristics in the year prior to a gold medal endurance performance." PLoS One **9**(7): e101796.

Turagam, M. K., P. Velagapudi and A. G. Kocheril (2012). "Atrial fibrillation in athletes." Am J Cardiol **109**(2): 296-302.

Watson, T. A., R. Callister, R. D. Taylor, D. W. Sibbritt, L. K. MacDonald-Wicks and M. L. Garg (2005). "Antioxidant restriction and oxidative stress in short-duration exhaustive exercise." Med Sci Sports Exerc **37**(1): 63-71.

PROSJEKT BESKRIVELSE – Antioksidantrik kost og høydetrening

Effekten av økt inntak av antioksidantrike matvarer på treningsadaptasjon, redoksbalanse og inflammasjon blant toppidrettsutøvere under en treningssamling i moderat høyde (2300m)

Koivisto A[1], Siv Kjølsrud Bøhn[2], Ingvild Paur[2], Gøran Paulsen1, Espen Tønnesen[1], Ina Garthe[1], Truls Raastad[3],Rune Blomhoff[2]

[1]Olympiatoppen, Sognsveien 228, 0806 Oslo, [2]Institutt for medisinske basalfag, Det medisinske fakultet, Universitetet i Oslo, [3]Seksjon for fysisk prestasjonsevne, Norges idrettshøgskole, Oslo

Sammendrag

Idrettsutøvere, spesielt innen utholdenhetsidretter, har økt risiko for oksidativt stress- og inflammasjonsrelaterte sykdommer og skader. Under trening, og spesielt under høydeopphold, øker produksjonen av reaktive oksygen- og nitrogenforbindelser (RONS) samt inflammasjonsmarkører. Inntak av antioksidanttilskudd er blitt en vanlig ernæringsstrategi blant idrettsutøvere i den tro at man hindrer oksidativt stress og oksidativ skade. En forbigående økning av RONS er imidlertid nødvendig for å aktivere signalkaskader som initierer treningsadaptasjon. Inntak av antioksidanttilskudd har vist seg å hemme treningseffekter i flere uavhengige studier, muligens ved å forstyrre/redusere signalkaskadene som igangsettes av RONS. Det er imidlertid ikke kjent om et høyt inntak av antioksidantrike matvarer kan påvirke mengden av RONS, betennelsesmarkører og/eller treningsadaptasjon. Vi ønsker derfor å undersøke om et økt inntak av naturlige antioksidanter i form av frukt, grønnsaker og bær, i tråd med de offisielle norske kostrådene, kan påvirke antioksidantstatus, immunforsvar og treningsadaptasjon i forbindelse med høydetrening for norske eliteutøvere som skal til i OL 2016.

Bakgrunn

Reaktive oksygen- og nitrogenforbindelser (RONS) dannes kontinuerlig som et resultat av naturlig cellemetabolisme. Kroppens antioksidantforsvar kan vanligvis nøytralisere og uskadeliggjøre disse forbindelsene før cellenes lipider, proteiner og DNA blir skadet, men dersom det oppstår ubalanse mellom RONS og cellenes antioksidantkapasitet vil det medføre en tilstand av oksidativt stress. Mange kroniske livsstilsykdommer er forbundet med oksidativt stress (Hu 2003), og idrettsutøvere, spesielt innen utholdenhetsidretter, har økt risiko for oksidativt stress- og inflammasjonsrelaterte sykdommer og skader (Carlsen, Hem et al. 2011, Metz, Wustrack et al. 2012, Turagam, Velagapudi et al. 2012, Dean, Gettings et al. 2015, Elkington, Gleeson et al. 2015, Sugama, Suzuki et al. 2015). Under trening øker produksjon av RONS på en doseavhengig måte (Knez, Jenkins et al. 2014). Dette

kan resultere i forstyrret cellefunksjon og bidra til muskulær tretthet og muligens forsinket restitusjon (Powers and Jackson 2008). Antioksidanter, en fellesbetegnelse på molekyler som har evne til å beskytte andre molekyler fra å bli oksidert, har dermed potensial til å motvirke oksidativt stress. Derfor tror mange utøvere at det er nødvendig å innta store mengder antioksidanter i form av kosttilskudd, for å beskytte seg not skadelige effekter av trening. Til tross mulige negative effekter av oksidativt stress, er en forbigående økning av RONS et viktig signal for treningsadaptasjon. RONS synes å aktivere flere signalmolekyler, blant annet PGC-1α som er en sentral regulator for mitokondriell biogenese (Powers, Duarte et al. 2010). Tidligere studier har vist at høye doser av enkelte antioksidanter fra kosttilskudd kan redusere responsen til utholdenhetstrening (Gomez-Cabrera, Domenech et al. 2008, Ristow, Zarse et al. 2009, Paulsen, Cumming et al. 2014) Derfor spekuleres det at antioksidanttilskudd hindrer treningsadaptasjon ved å forstyrre signalkaskadene som igangsettes av RONS.

Det er ikke kjent om et høyt inntak av naturlige antioksidanter via inntak av frukt og grønnsaker kan ha en liknende uønsket effekt på treningsrespons. Et høyt inntak av frukt og grønnsaker, som er naturlig rike på antioksidanter, beskytter mot en rekke sykdommer assosiert med oksidativt stress. I tillegg har et antioksidantfattig kosthold, dvs lavt inntak av frukt og grønnsaker, blant idrettsutøvere blitt assosiert med økte nivåer av systemiske betennelsesmarkører (Watson, Callister et al. 2005, Plunkett, Callister et al. 2010). Siden frukt og grønnsaker er rike på antioksidanter har det vært foreslått at antioksidantene fra mat gir ekstra beskyttelse mot oksidering i kroppen. Det er imidlertid ikke mulig å si om det er antioksidantene som gjør oss mer beskyttet mot sykdom siden frukt og grønnsaker inneholder ti-tusenvis av ulike plantekjemikalier (fytokjemikalier). De fleste fytokjemikalier har antioksidant egenskaper, men de har også andre egenskaper som kan ha ulike effekter i kroppen. Blant annet kan andre egenskaper ved fytokjemikaliene være viktige for å optimalisere kroppens egne forsvarssystemer (Bøhn, Myhrstad et al. 2010).

Ved Olympiatoppens ernæringsavdeling frarådes idrettsutøverne å bruke høye doser av enkelte antioksidanttilskudd. Generelt anbefales det å øke inntaket av antioksidantrike matvarer i perioder med stor treningsbelastning som strategi for å unngå negative effekter av oksidativ stress. Norske helsemyndigheter anbefaler minst fem porsjoner frukt, bær og grønnsaker per dag (500g) (Helsedirektoratet, 2014). I en kostholdsundersøkelse blant idrettsutøvere på høydetrening i juni 2015 ble det avdekket at inntaket av frukt og grønnsaker ligger langt under helsemyndighetenes anbefalinger. I tillegg er det usikkert om fem om dagen gir et tilstrekkelig inntak av antioksidanter for utholdenhetsutøvere i de mest krevende treningssyklusene.

For å teste om naturlige antioksidanter fra plantemat har effekt på treningsadaptasjon ønsker vi å utføre en klinisk kontrollert studie på idrettsutøvere som blir eksponert for ekstra høye nivåer av oksidativt stress ved treningsopphold i høyden (2300 moh). Høydeeksponering, som gir redusert oksygentilgjengelighet (hypoksi), er tidligere vist å øke det oksidative stressnivået (Pialoux, Mounier et al. 2009, Pialoux, Mounier et al. 2009). Undersøkelser som har benyttet "live high – train low" -modellen (trene på 1200m og bo på 2500-3000m simulert høyde) blant toppidrettsutøvere har vist økt oksidativt stress. Samtidig har antioksidantkapasiteten i serum blitt redusert når varighet av høydeeksponering har passert 18 dager (Pialoux, Mounier et al. 2009, Pialoux, Mounier et al. 2009). Gitt at eksponering til høyde og større treningsbelastning øker oksidativt stress er det mulig at idrettsutøvernes redoksbalanse kan endres under treningsopphold i høyden («live high- train high» metoden). Høydetrening vil også belaste kroppens immunforsvar. Tidligere studier har vist at trening i hypoksi øker utskillelse av enkelte cytokiner og fører til en forbigående immunosuppresjon (Mazzeo 2005). I tillegg til at det er ukjent hvordan et antioksidantrikt kosthold vil påvirke treningsadaptasjon, antioksidantbalanse, immunforsvar og genekspresjon under høydetrening. Det finnes heller ikke data på toppidrettsutøveres antioksidantinntak under treningssamling i høyden. Det er også mangel på referanseverdier for biomarkører for antioksidantkapasitet og oksidativt stress hos toppidrettsutøvere (Lewis, Howatson et al. 2015).

Formål

Formålet med studien er å undersøke om et antioksidantrikt kosthold kan påvirke helseeffekter av høydetrening hos toppidrettsutøvere ved å optimalisere mellommåltider i intervensjonsgruppen mht inntak av antioksidantrike matvarer i forbindelse med en 3-ukers treningssamling i moderat høyde (2300 m). Vi ønsker spesielt å kartlegge inntak av antioksidanter, oksidativt stress og antioksidant kapasitet før, under og etter denne høydesamlingen. Videre vil vi studere effekten av et antioksidantrikt kosthold på redoksbalanse, inflammasjonsstatus og treningsadaptasjon under en 3-ukers høydetreningssamling. Med spesiell fokus på stress-respons (kroppens egne forsvarsystemer) vil vi studere hvordan et høydeopphold påvirker genekspresjonsprofil og om intervensjonen påvirker disse forsvarssystemene sammenliknet med kontroll.

Hypoteser

1. Tilpasningen til høyde med økt hemoglobinmasse vil ikke påvirkes av økt inntak av antioksidantrike mellommåltider.
2. Idrettslig prestasjonsevne vil ha en mer positiv utvikling under høydetreningssamlingen med inntak av antioksidantrike mellommåltider, fordi muskulatur og sirkulasjonssystemet bedre vil tåle kombinasjonen av hypoksi og stort treningsvolum.
3. Idrettsutøvere har suboptimalt inntak av frukt, bær og grønnsaker (< 500g pr dag) i lavland og i høyden.
4. Høydeeksponeringen kombinert med stort treningsvolum vil øke det oksidativt stresset og redusere antioksidantkapasiteten gjennom høydeoppholdet. Dette vil bl.a. resultere i økte nivåer av betennelsesmarkører.
5. Daglige inntak av antioksidantrike mellommåltider vil øke sirkulerende nivåer av utvalgte antioksidanter og begrense økningen i markører for oksidativt stress og betennelse.
6. Høydetrening medfører endringer i genekspresjonsprofilen i blodceller, spesielt gener som er involvert i stress respons. Endringer i genekspresjonsprofil som følger av høydetrening er forskjellig mellom gruppene.

Endepunkter

- Virkning av et høydeopphold: f.eks hemoglobinmasse og blodvolum, maksimalt oksygenopptak (VO2maks), laktatprofil, prestasjon i idrettsspesifikke tester
- Antropometri: f.eks kroppsvekt, lean body mass (LBM), fettmasse (FM), bentetthet (BMD)
- Kosthold: f.eks energi, makronæringsstoffer, mikronæringsstoffer, antall porsjoner frukt, grønnsaker og bær, samt totalt antioksidantinntak (mmol)
- Compliance: biomarkører for frukt og grønnsaksinntak, (f. eks karotenoider)
- Betennelsesmarkører : f.eks IL-1, IL-6, TNFα
- Genekspresjon: Whole genome gene expression
- Antioksidant status:
  - Oksidativ stress biomarkører: f.eks lipid peroksidasjon (f.eks 8-isoPGF2α).
  - Antioksidantkapasitet: f.eks ferric reducing ability of plasma (FRAP)

    Total oksidativ stress: f.eks dROM
- Antioksidanter i plasma: f.eks karotenoider, flavonoider
- Andre data fra rutinemessig medisinsk screening av utøverne (f.eks. østradiol, testosteron, crp, kortisol, hb, ferritin, hematokrit, vitamin D, vitamin E, B12, folat, kolesterol, HDL, LDL)

Deltakere

Inklusjonskriterier

- Landslagsutøvere, menn og kvinner (18-42 år) fra padling, roing, sykling, svømming og triatlon som skal delta på en høydesamling for OL 2016. Aktuelle utøvere i utholdenhetsidretter vil bli forespurt om å delta.

Eksklusjonskriterier

- Allergier mot de aktuelle matvarer der det ikke er mulig å erstatte matvarer med liknende antioksidantrike produkter i samråd med deltakeren.
- Bruk av medisiner som kan påvirke treningen og prestasjonstester.
- Skader som kan påvirke trening og prestasjonstester.

Rekruttering

Deltakere vil bli rekruttert fra potensielle OL 2016-utøvere i utholdenhetsidretter. Utøverne vil bli randomisert til en intervensjonsgruppe eller kontrollgruppe stratifisert for idrett og kjønn.

Styrkeberegninger

Basert på en effektstørrelse på 1.0, med to-sidig signifikansnivå på 5% og 80% power trenger vi 17 idrettsutøvere i hver gruppe. Estimert endring i effektstørrelse er basert på variasjonen i prestasjonstestene i 2000 m roing på roergometer der varaiasjonen fra dag til dag er på 1.3 % (SD) under ellers like forhold. En endring i prestasjon på 1.3% tilsvarer ca. 5 sekunder forbedring på 2000 m roing; en bedring som kan skille medaljevinnere i et OL. Samme relative endringer er ventet i prestasjon på de andre idrettene representert i forsøket. Dersom vi beregner en dropout % på 10 vil vi trenge 19 i hver gruppe for å påvise signifikante forskjeller mellom gruppene. Vi vil invitere alle aktuelle utøvere til å delta (n=45) fordi vi regner med at 85% samtykker til å delta.

Tidsakse

Deltakerne vil bli rekruttert fire uker før høydeoppholdet i slutten av september 2015. Intervensjonen og høydeoppholdet i Spania vil avsluttes 11.november 2015. Alle deltakere vil gjennomgå en generell medisinsk undersøkelse og få kartlagt kliniske parametere tre uker i forkant av høydeopphold. I tillegg kartlegges hemoglobinmasse og kroppssammensetning én uke i forkant og i etterkant av høydeoppholdet. Det vil bli foretatt repeterte registreringer av kosthold, antropometri og prestasjon, samt blodprøvetaking, før, under og etter høydeoppholdet (Figur 1).

Figur 1. Tidslinje av studieforløpet.

UKE -3	UKE -2	UKE -1	UKE 1	UKE 2	UKE 3	UKE 4
Medisinsk us		DXA	HØYDEOPPHOLD (2300m)			DXA
Jernstatus		Blodvolum<br>VO2 max<br>Laktat profil	INTERVENSJON<br>Daglig inntak av antioksidantrike matvarer			Blodvolum<br>VO2 max<br>Laktat profil
		Prestasjonstester	Prestasjonstester		Prestasjonstester	Prestasjonstester
		Blodprøver	Blodprøver		Blodprøver	Blodprøver
		Urinprøve	Urinprøve		Urinprøve	Urinprøve
24 h recall		2 x 24 h recall	24 h recall	24 h recall	24 h recall	

Metoder

Intervensjonen

Intervensjonens hensikt er å optimalisere mellommåltider hos idrettsutøvere under høydeopphold med hensyn på inntak av antioksidantrike matvarer. Intervensjonsgruppen vil motta matvarer med høyt innhold av antioksidanter (bl.a. bær, nøtter, mørk sjokolade, grønnsaker og frukt som har et naturlig høyt nivå av antioksidanter (Carlsen, Halvorsen et al. 2010). Kontrollgruppen vil få matvarer som vanligvis inntas som mellommåltider under høydeopphold. Mellommåltidene til gruppene vil bli matchet med hensyn til kaloriinnhold (ca 1000 kcal), og vil bli utdelt en gang om dagen til den enkelte deltaker. Utøverne i begge grupper oppfordres ellers til å holde stabil kroppsvekt (unngå avvik i kroppsvekt > 2 %) gjennom hele høydeoppholdet.

Mellommåltider intervensjonsgruppen (daglige)

Målet er å doble antioksidantinntaket til utøverne i intervensjonsgruppen ved å tilby ulike antioksidantrike mellommåltider. Intervensjonen som er beskrevet nedenfor er et eksempel. Andre vanlige antioksidantrike matvarer kan også inngå.

- 3 stk frukt/bær/grønnsak smoothie valgt fra følgende produkter (450 kcal):
  - Bama, Turn 250 ml (71 kcal/100ml): 44 % druejuice, 30 % bananpuré, 17 % granateplejuice og 9 % solbærjuice
  - Bama, Slide 250 ml (47 kcal/100ml): 38 % eplejuice, 20 % gulrotpuré, 20 % mangopuré 20 %, 17 % eplepuré og 5% pasjonsfruktpuré
  - Bama, Spin 250 ml (62 kcal/100ml): 38 % eplejuice, 21 % rød druejuice, 20 % bananpuré, 8 % blåbærpuré, 8 % solbærjuice og 5 % eplepuré
- 1 dl (40 g) valnøtter (270 kcal)
- 1 dl (50g) blanding av tørket frukt og bær; tranebær, gojibær, aprikos (Rema1000 og Bama) (140 kcal)
- 40g mørk sjokolade (70%), utvalg av Freia Premium sjokolader i smaksvarianter; vanlig, pekan og havsalt, appelsin, mandel, hasselnøtt (240kcal)

Kontrollgruppen vil innta daglig:

- 220 ml TINE milkshake i smaksvarianter; jordbær og banan eller kakao (160 kcal)
- 330ml TINE YT restitusjonsdrikk i smaksvarianter; jordbær og banan eller kakao (220 kcal)
- 4 Bixit kjeks (60 g), eller annen type søte kjeks uten fyll (290 kcal)
- 1 dl (30g) Ritz salte kjeks /saltstenger (120 kcal)
- 50g Freia hvit sjokolade (270 kcal)

Antropometri

Standardiserte DXAmåling (dual energy x-ray; iDXA; GE Healthcare) tas før og etter treningsleir for å måle muskelmasse (LBM), fettmasse (FM) og bentetthet (BMD). Kroppsvekt med minst mulig bekledning før frokost registreres daglig og utøverne oppfordres til å holde stabil kroppsvekt (unngå avvik i kroppsvekt > 2%) gjennom hele høydeleiren.

Kostregistreringsmetoder

Det blir gjennomført fire 24 h recalls i en intervjusetting før høydeoppholdet, og tre ganger under høydeoppholdet av samme ernæringsfysiolog. 24 h recalls vil være separert med minst 2 dager. Intervjuguide for 24 h recall følger i eget vedlegg.

Blodprøver

Fastende blodprøver med venepunksjon samles før og etter høydesamling samt på to testdager i løpet av høydeoppholdet. I tillegg tas det ukentlige blodprøver fra fingertuppen under høydesamlingen. Det brukes Dried Blood Spot metode som muliggjør målinger av diverse biomarkører på en mindre invasiv måte. Laboratoriet ved UiO vil analysere blodprøvene mht biomarkører som f.eks. oksidativ skade, inflammasjonsstatus, antioksidantstatus og effekt på gen-ekspresjon i blodceller. Protokoll for blodprøvetaking og håndtering følger i eget vedlegg. Helblod gentranskripsjonsanalyser, low density gene arrays og/eller RT-PCR vil bli utført på blodceller som blir innsamlet ved de ulike tidspunktene.

Blodvolums målinger

Hemoglobinmasse, plasmavolum og totalt blodvolum vil bli estimert ved CO-rebreathing-metoden etter Burge og Skinner (Burge and Skinner 1995). Denne prosedyren vil gjennomføres to ganger før og etter intervensjonsperioden i høyden.

Fysiologiske tester og prestasjonstester

Deltakerne i denne studien er toppidrettsutøvere med tilknytting til Olympiatoppen. Alle utøverne har således erfaring med testene beskrevet under.

Prestasjonstesten og maksimalt oksygenopptak (VO2maks) vil kun testes ved Olympiatoppen (i lavlandet) før og etter intervensjonsperioden i høyden. Laktatprofiltesten (uten oksygenopptaksmålinger) vil også gjennomføres i høyden: 4-5 dager etter deltakerne kommer til høyden og 2-3 dager før hjemreise.

Laktatprofiltest

Deltakerne vil i et idrettsspesifikt ergometer/element (sykkel-, ro- og padleapparat, eller vann) gjennomføre en laktatprofiltest. Dette gjennomføres etter standard prosedyre ved Olympiatoppen

(Stoa, Storen et al. 2010). Etter 10 min oppvarming på 60-70 % av HF-maks, gjennomføres 4 til 6 intervalldrag på 5 minutters med økende intensitet. Testen avsluttes når blodlaktatverdien er 1,5 mmol høyere enn laktatverdien på de to første intervalldragene. Mellom hvert intervalldrag er det en pause på 30 sekunder.

Laktat måles i helblod tatt ved fingerstikk. Fingertuppen vaskes og tørkes før hvert stikk. Oksygenopptak[1] (se under) og hjertefrekvens registreres underveis på hvert intervalldrag.

Maksimal oksygenopptak (VO2maks)

I forlengelsen av laktatprofiltesten gjennomføres det en VO2maks-test etter standard prosedyre ved Olympiatoppen (Tonnessen, Haugen et al. 2015). Testen starter på ett intensitetsnivå under anaerob terskel, og intensiteten økes hvert minutt. Testen gjennomføres til utmattelse, og varer typisk i 4-6 min. Svømmere vil måle VO2maks ved løping i motbakke.

Prestasjonstest

I et idrettsspesifikt ergometer/element vil deltakerne gjennomføre distanse test, der målet er å produsere så høy gjennomsnitts-watt/hastighet som mulig. Varigheten vil variere fra ca 2 min til 15 min avhengig av deltakerens idrettsgren.

- Sykling: 10 000 m på sykkelrulle
- Roing: 2000 m i Concept2-roapparat
- Padling: 1000 m Concept2-padleapparat
- Svømming: 200 m i vann (egen svømmeart)

Hjertefrekvens måles underveis, mens laktat måles 1 min etter avsluttet test.

Dokumentasjon av trening

Før, under og etter høydeoppholdet registrerer utøverne utført trening i Olympiatoppens treningsdagbok. I treningsdagboken registreres varigheten og intensiteten på treningen, samt treningsform og bevegelsesform. Treningen dokumenteres på samme måte som tidligere beskrevet beskrevet (Tonnessen, Sylta et al. 2014).

Hjertefrekvens måles underveis, mens laktat måles 1 min etter avsluttet test.

---

[1] Oksygenopptak vil ikke måles hos svømmere.

Oppmøteoversikt

Visitt 1: Målingene foregår på helseavdeling på Olympiatoppen i Oslo.

- Generell medisinsk undersøkelse
- Blodprøver (venøs)
- Måling av hemoglobinmasse
- 24 timers recall (se vedlegg)

Visitt 2: Målingene foregår på helseavdeling og testlab på Olympiatoppen i Oslo.

- Fastende DXA måling (på NIH)
- Måling av hemoglobinmasse
- 24 h recall
- Laktatprofil
- VO2maks

Visitt 3: Målingene foregår på helseavdeling og testlab på Olympiatoppen i Oslo.

- Fastende blodprøver (venøs og fingertupp) og urinprøve
- Blodprøver etter frokost (venøs og fingertupp)
- Prestasjonstester
- Blodprøver etter prestasjonstester (venøs og fingertupp)
- 24 h recall

Visitt 4: Målingene foregår i Centro de alto rendimento i Sierra Nevada, Spania.

- Fastende blodprøver (venøs og fingertupp) og urinprøve
- Blodprøver etter frokost (venøs og fingertupp)
- Laktatprofil
- Blodprøver etter laktatprofil (venøs og fingertupp)
- 24 h recall

Visitt 5: Målingene foregår i Centro de alto rendimento i Sierra Nevada, Spania.

- Fastende blodprøver (venøs og fingertupp) og urinprøve
- Blodprøver etter frokost, rett før laktatprofil (venøs og fingertupp)
- Laktatprofil
- Blodprøver etter laktatprofil (venøs og fingertupp)
- 24 h recall

Visitt 6: Målingene foregår på helseavdeling og testlab på Olympiatoppen i Oslo.

- Fastende blodprøver (venøs og fingertupp) og urinprøve
- Måling av hemoglobinmasse
- Blodprøver etter frokost (venøs og fingertupp)
- Prestasjonstester
- Blodprøver etter prestasjonstester (venøs og fingertupp)

Visitt 7: Målingene foregår på helseavdeling og testlab på Olympiatoppen i Oslo

- Fastende DXA måling
- Måling av hemoglobinmasse
- 24 h recall
- Laktatprofil
- VO2maks

Referanser

Burge, C. M. and S. L. Skinner (1995). "Determination of hemoglobin mass and blood volume with CO: evaluation and application of a method." J Appl Physiol (1985) **79**(2): 623-631.

Bøhn, S. K., M. C. Myhrstad, M. Thoresen, M. Holden, A. Karlsen, S. H. Tunheim, I. Erlund, M. Svendsen, I. Seljeflot, J. O. Moskaug, A. K. Duttaroy, P. Laake, H. Arnesen, S. Tonstad, A. Collins, C. A. Drevon and R. Blomhoff (2010). "Blood cell gene expression associated with cellular stress defense is modulated by antioxidant-rich food in a randomised controlled clinical trial of male smokers." BMC Med **8**: 54.

Carlsen, K. H., E. Hem and T. Stensrud (2011). "Asthma in adolescent athletes." Br J Sports Med **45**(16): 1266-1271.

Carlsen, M. H., B. L. Halvorsen, K. Holte, S. K. Bohn, S. Dragland, L. Sampson, C. Willey, H. Senoo, Y. Umezono, C. Sanada, I. Barikmo, N. Berhe, W. C. Willett, K. M. Phillips, D. R. Jacobs, Jr. and R. Blomhoff (2010). "The total antioxidant content of more than 3100 foods, beverages, spices, herbs and supplements used worldwide." Nutr J **9**: 3.

Dean, B. J., P. Gettings, S. G. Dakin and A. J. Carr (2015). "Are inflammatory cells increased in painful human tendinopathy? A systematic review." Br J Sports Med.

Elkington, L. J., M. Gleeson, D. B. Pyne, R. Callister and L. G. Wood (2015). Inflammation and Immune Function: Can Antioxidants Help the Endurance Athlete? Antioxidants in Sport Nutrition. M. Lamprecht. Boca Raton FL, 2015 by Taylor & Francis Group, LLC.

Gomez-Cabrera, M. C., E. Domenech, M. Romagnoli, A. Arduini, C. Borras, F. V. Pallardo, J. Sastre and J. Vina (2008). "Oral administration of vitamin C decreases muscle mitochondrial biogenesis and hampers training-induced adaptations in endurance performance." Am J Clin Nutr **87**(1): 142-149.

Hu, F. B. (2003). "Plant-based foods and prevention of cardiovascular disease: an overview." Am J Clin Nutr **78**(3 Suppl): 544S-551S.

Knez, W. L., D. G. Jenkins and J. S. Coombes (2014). "The effect of an increased training volume on oxidative stress." Int J Sports Med **35**(1): 8-13.

Lewis, N. A., G. Howatson, K. Morton, J. Hill and C. R. Pedlar (2015). "Alterations in redox homeostasis in the elite endurance athlete." Sports Med **45**(3): 379-409.

Mazzeo, R. S. (2005). "Altitude, exercise and immune function." Exerc Immunol Rev **11**: 6-16.

Metz, L. N., R. Wustrack, A. F. Lovell and A. J. Sawyer (2012). "Infectious, inflammatory, and metabolic diseases affecting the athlete's spine." Clin Sports Med **31**(3): 535-567.

Paulsen, G., K. T. Cumming, G. Holden, J. Hallen, B. R. Ronnestad, O. Sveen, A. Skaug, I. Paur, N. E. Bastani, H. N. Ostgaard, C. Buer, M. Midttun, F. Freuchen, H. Wiig, E. T. Ulseth, I. Garthe, R. Blomhoff, H. B. Benestad and T. Raastad (2014). "Vitamin C and E supplementation hampers cellular adaptation to endurance training in humans: a double-blind, randomised, controlled trial." J Physiol **592**(Pt 8): 1887-1901.

Pialoux, V., R. Mounier, J. V. Brugniaux, E. Rock, A. Mazur, J. P. Richalet, P. Robach, J. Coudert and N. Fellmann (2009). "Thirteen days of "live high-train low" does not affect prooxidant/antioxidant balance in elite swimmers." Eur J Appl Physiol **106**(4): 517-524.

Pialoux, V., R. Mounier, E. Rock, A. Mazur, L. Schmitt, J. P. Richalet, P. Robach, J. Brugniaux, J. Coudert and N. Fellmann (2009). "Effects of the 'live high-train low' method on prooxidant/antioxidant balance on elite athletes." Eur J Clin Nutr **63**(6): 756-762.

Plunkett, B. A., R. Callister, T. A. Watson and M. L. Garg (2010). "Dietary antioxidant restriction affects the inflammatory response in athletes." Br J Nutr **103**(8): 1179-1184.

Powers, S. K., J. Duarte, A. N. Kavazis and E. E. Talbert (2010). "Reactive oxygen species are signalling molecules for skeletal muscle adaptation." Exp Physiol **95**(1): 1-9.

Powers, S. K. and M. J. Jackson (2008). "Exercise-induced oxidative stress: cellular mechanisms and impact on muscle force production." Physiol Rev **88**(4): 1243-1276.

Ristow, M., K. Zarse, A. Oberbach, N. Kloting, M. Birringer, M. Kiehntopf, M. Stumvoll, C. R. Kahn and M. Bluher (2009). "Antioxidants prevent health-promoting effects of physical exercise in humans." Proc Natl Acad Sci U S A **106**(21): 8665-8670.

Stoa, E. M., O. Storen, E. Enoksen and F. Ingjer (2010). "Percent utilization of VO2 max at 5-km competition velocity does not determine time performance at 5 km among elite distance runners." J Strength Cond Res **24**(5): 1340-1345.

Sugama, K., K. Suzuki, K. Yoshitani, K. Shiraishi, S. Miura, H. Yoshioka, Y. Mori and T. Kometani (2015). "Changes of thioredoxin, oxidative stress markers, inflammation and muscle/renal damage following intensive endurance exercise." Exerc Immunol Rev **21**: 130-142.

Tonnessen, E., T. A. Haugen, E. Hem, S. Leirstein and S. Seiler (2015). "Maximal Aerobic Capacity in the Winter Olympic Endurance Disciplines: Olympic Medal Benchmarks for the Time Period 1990-2013." Int J Sports Physiol Perform.

Tonnessen, E., O. Sylta, T. A. Haugen, E. Hem, I. S. Svendsen and S. Seiler (2014). "The road to gold: training and peaking characteristics in the year prior to a gold medal endurance performance." PLoS One **9**(7): e101796.

Turagam, M. K., P. Velagapudi and A. G. Kocheril (2012). "Atrial fibrillation in athletes." Am J Cardiol **109**(2): 296-302.

Watson, T. A., R. Callister, R. D. Taylor, D. W. Sibbritt, L. K. MacDonald-Wicks and M. L. Garg (2005). "Antioxidant restriction and oxidative stress in short-duration exhaustive exercise." Med Sci Sports Exerc **37**(1): 63-71.
