# Supplementary material for: Effects of antioxidant-rich foods on altitude-induced oxidative stress and inflammation in elite endurance athletes: A randomized controlled trial
Source: PLoS One. 2019 Jun 13;14(6):e0217895. doi: 10.1371/journal.pone.0217895 (PMC6563980; doi:10.1371/journal.pone.0217895)
Supplement: S3 Text — (DOCX) [file pone.0217895.s009.docx]

**PROSJEKT BESKRIVELSE – Antioksidantrik kost og høydetrening**

**Effekten av økt inntak av antioksidantrike matvarer på treningsadaptasjon, redoksbalanse og inflammasjon blant toppidrettsutøvere under en treningssamling i moderat høyde (2300m)**

Koivisto A^1^, Siv Kjølsrud Bøhn^2^, Ingvild Paur^2^, Gøran Paulsen1, Espen Tønnesen^1^, Ina Garthe^1^, Truls Raastad^3^,Rune Blomhoff^2^

^1^Olympiatoppen, Sognsveien 228, 0806 Oslo, ^2^Institutt for medisinske basalfag, Det medisinske fakultet, Universitetet i Oslo, ^3^Seksjon for fysisk prestasjonsevne, Norges idrettshøgskole, Oslo

**Sammendrag**

Idrettsutøvere, spesielt innen utholdenhetsidretter, har økt risiko for oksidativt stress- og inflammasjonsrelaterte sykdommer og skader. Under trening, og spesielt under høydeopphold, øker produksjonen av reaktive oksygen- og nitrogenforbindelser (RONS) samt inflammasjonsmarkører. Inntak av antioksidanttilskudd er blitt en vanlig ernæringsstrategi blant idrettsutøvere i den tro at man hindrer oksidativt stress og oksidativ skade. En forbigående økning av RONS er imidlertid nødvendig for å aktivere signalkaskader som initierer treningsadaptasjon. Inntak av antioksidanttilskudd har vist seg å hemme treningseffekter i flere uavhengige studier, muligens ved å forstyrre/redusere signalkaskadene som igangsettes av RONS. Det er imidlertid ikke kjent om et høyt inntak av antioksidantrike matvarer kan påvirke mengden av RONS, betennelsesmarkører og/eller treningsadaptasjon. Vi ønsker derfor å undersøke om et økt inntak av naturlige antioksidanter i form av frukt, grønnsaker og bær, i tråd med de offisielle norske kostrådene, kan påvirke antioksidantstatus, immunforsvar og treningsadaptasjon i forbindelse med høydetrening for norske eliteutøvere som skal til i OL 2016.

**Bakgrunn**

Reaktive oksygen- og nitrogenforbindelser (RONS) dannes kontinuerlig som et resultat av naturlig cellemetabolisme. Kroppens antioksidantforsvar kan vanligvis nøytralisere og uskadeliggjøre disse forbindelsene før cellenes lipider, proteiner og DNA blir skadet, men dersom det oppstår ubalanse mellom RONS og cellenes antioksidantkapasitet vil det medføre en tilstand av oksidativt stress. Mange kroniske livsstilsykdommer er forbundet med oksidativt stress (Hu 2003), og idrettsutøvere, spesielt innen utholdenhetsidretter, har økt risiko for oksidativt stress- og inflammasjonsrelaterte sykdommer og skader (Carlsen, Hem et al. 2011, Metz, Wustrack et al. 2012, Turagam, Velagapudi et al. 2012, Dean, Gettings et al. 2015, Elkington, Gleeson et al. 2015, Sugama, Suzuki et al. 2015). Under trening øker produksjon av RONS på en doseavhengig måte (Knez, Jenkins et al. 2014). Dette kan resultere i forstyrret cellefunksjon og bidra til muskulær tretthet og muligens forsinket restitusjon (Powers and Jackson 2008). Antioksidanter, en fellesbetegnelse på molekyler som har evne til å beskytte andre molekyler fra å bli oksidert, har dermed potensial til å motvirke oksidativt stress. Derfor tror mange utøvere at det er nødvendig å innta store mengder antioksidanter i form av kosttilskudd, for å beskytte seg not skadelige effekter av trening. Til tross mulige negative effekter av oksidativt stress, er en forbigående økning av RONS et viktig signal for treningsadaptasjon. RONS synes å aktivere flere signalmolekyler, blant annet PGC-1α som er en sentral regulator for mitokondriell biogenese (Powers, Duarte et al. 2010). Tidligere studier har vist at høye doser av enkelte antioksidanter fra kosttilskudd kan redusere responsen til utholdenhetstrening (Gomez-Cabrera, Domenech et al. 2008, Ristow, Zarse et al. 2009, Paulsen, Cumming et al. 2014) Derfor spekuleres det at antioksidanttilskudd hindrer treningsadaptasjon ved å forstyrre signalkaskadene som igangsettes av RONS.

Det er ikke kjent om et høyt inntak av naturlige antioksidanter via inntak av frukt og grønnsaker kan ha en liknende uønsket effekt på treningsrespons. Et høyt inntak av frukt og grønnsaker, som er naturlig rike på antioksidanter, beskytter mot en rekke sykdommer assosiert med oksidativt stress. I tillegg har et antioksidantfattig kosthold, dvs lavt inntak av frukt og grønnsaker, blant idrettsutøvere blitt assosiert med økte nivåer av systemiske betennelsesmarkører (Watson, Callister et al. 2005, Plunkett, Callister et al. 2010). Siden frukt og grønnsaker er rike på antioksidanter har det vært foreslått at antioksidantene fra mat gir ekstra beskyttelse mot oksidering i kroppen. Det er imidlertid ikke mulig å si om det er antioksidantene som gjør oss mer beskyttet mot sykdom siden frukt og grønnsaker inneholder ti-tusenvis av ulike plantekjemikalier (fytokjemikalier). De fleste fytokjemikalier har antioksidant egenskaper, men de har også andre egenskaper som kan ha ulike effekter i kroppen. Blant annet kan andre egenskaper ved fytokjemikaliene være viktige for å optimalisere kroppens egne forsvarssystemer (Bøhn, Myhrstad et al. 2010).

Ved Olympiatoppens ernæringsavdeling frarådes idrettsutøverne å bruke høye doser av enkelte antioksidanttilskudd. Generelt anbefales det å øke inntaket av antioksidantrike matvarer i perioder med stor treningsbelastning som strategi for å unngå negative effekter av oksidativ stress. Norske helsemyndigheter anbefaler minst fem porsjoner frukt, bær og grønnsaker per dag (500g) (Helsedirektoratet, 2014). I en kostholdsundersøkelse blant idrettsutøvere på høydetrening i juni 2015 ble det avdekket at inntaket av frukt og grønnsaker ligger langt under helsemyndighetenes anbefalinger. I tillegg er det usikkert om fem om dagen gir et tilstrekkelig inntak av antioksidanter for utholdenhetsutøvere i de mest krevende treningssyklusene.

For å teste om naturlige antioksidanter fra plantemat har effekt på treningsadaptasjon ønsker vi å utføre en klinisk kontrollert studie på idrettsutøvere som blir eksponert for ekstra høye nivåer av oksidativt stress ved treningsopphold i høyden (2300 moh). Høydeeksponering, som gir redusert oksygentilgjengelighet (hypoksi), er tidligere vist å øke det oksidative stressnivået (Pialoux, Mounier et al. 2009, Pialoux, Mounier et al. 2009). Undersøkelser som har benyttet “live high – train low” -modellen (trene på 1200m og bo på 2500-3000m simulert høyde) blant toppidrettsutøvere har vist økt oksidativt stress. Samtidig har antioksidantkapasiteten i serum blitt redusert når varighet av høydeeksponering har passert 18 dager (Pialoux, Mounier et al. 2009, Pialoux, Mounier et al. 2009). Gitt at eksponering til høyde og større treningsbelastning øker oksidativt stress er det mulig at idrettsutøvernes redoksbalanse kan endres under treningsopphold i høyden («live high- train high» metoden). Høydetrening vil også belaste kroppens immunforsvar. Tidligere studier har vist at trening i hypoksi øker utskillelse av enkelte cytokiner og fører til en forbigående immunosuppresjon (Mazzeo 2005). I tillegg til at det er ukjent hvordan et antioksidantrikt kosthold vil påvirke treningsadaptasjon, antioksidantbalanse, immunforsvar og genekspresjon under høydetrening. Det finnes heller ikke data på toppidrettsutøveres antioksidantinntak under treningssamling i høyden. Det er også mangel på referanseverdier for biomarkører for antioksidantkapasitet og oksidativt stress hos toppidrettsutøvere (Lewis, Howatson et al. 2015).

# Formål

Formålet med studien er å undersøke om et antioksidantrikt kosthold kan påvirke helseeffekter av høydetrening hos toppidrettsutøvere ved å optimalisere mellommåltider i intervensjonsgruppen mht inntak av antioksidantrike matvarer i forbindelse med en 3-ukers treningssamling i moderat høyde (2300 m). Vi ønsker spesielt å kartlegge inntak av antioksidanter, oksidativt stress og antioksidant kapasitet før, under og etter denne høydesamlingen. Videre vil vi studere effekten av et antioksidantrikt kosthold på redoksbalanse, inflammasjonsstatus og treningsadaptasjon under en 3-ukers høydetreningssamling. Med spesiell fokus på stress-respons (kroppens egne forsvarsystemer) vil vi studere hvordan et høydeopphold påvirker genekspresjonsprofil og om intervensjonen påvirker disse forsvarssystemene sammenliknet med kontroll.

## Hypoteser

1. Tilpasningen til høyde med økt hemoglobinmasse vil ikke påvirkes av økt inntak av antioksidantrike mellommåltider.
2. Idrettslig prestasjonsevne vil ha en mer positiv utvikling under høydetreningssamlingen med inntak av antioksidantrike mellommåltider, fordi muskulatur og sirkulasjonssystemet bedre vil tåle kombinasjonen av hypoksi og stort treningsvolum.
3. Idrettsutøvere har suboptimalt inntak av frukt, bær og grønnsaker (< 500g pr dag) i lavland og i høyden.
4. Høydeeksponeringen kombinert med stort treningsvolum vil øke det oksidativt stresset og redusere antioksidantkapasiteten gjennom høydeoppholdet. Dette vil bl.a. resultere i økte nivåer av betennelsesmarkører.
5. Daglige inntak av antioksidantrike mellommåltider vil øke sirkulerende nivåer av utvalgte antioksidanter og begrense økningen i markører for oksidativt stress og betennelse.
6. Høydetrening medfører endringer i genekspresjonsprofilen i blodceller, spesielt gener som er involvert i stress respons. Endringer i genekspresjonsprofil som følger av høydetrening er forskjellig mellom gruppene.

## Endepunkter

- Virkning av et høydeopphold: f.eks hemoglobinmasse og blodvolum, maksimalt oksygenopptak (VO2maks), laktatprofil, prestasjon i idrettsspesifikke tester
- Antropometri: f.eks kroppsvekt, lean body mass (LBM), fettmasse (FM), bentetthet (BMD)
- Kosthold: f.eks energi, makronæringsstoffer, mikronæringsstoffer, antall porsjoner frukt, grønnsaker og bær, samt totalt antioksidantinntak (mmol)
- Compliance: biomarkører for frukt og grønnsaksinntak, (f. eks karotenoider)
- Betennelsesmarkører : f.eks IL-1, IL-6, TNFα
- Genekspresjon: Whole genome gene expression
- Antioksidant status:
  - Oksidativ stress biomarkører: f.eks lipid peroksidasjon (f.eks 8-isoPGF2α).
  - Antioksidantkapasitet: f.eks ferric reducing ability of plasma (FRAP)

Total oksidativ stress: f.eks dROM

- Antioksidanter i plasma: f.eks karotenoider, flavonoider
- Andre data fra rutinemessig medisinsk screening av utøverne (f.eks. østradiol, testosteron, crp, kortisol, hb, ferritin, hematokrit, vitamin D, vitamin E, B12, folat, kolesterol, HDL, LDL )

# Deltakere

## Inklusjonskriterier

- Landslagsutøvere, menn og kvinner (18-42 år) fra padling, roing, sykling, svømming og triatlon som skal delta på en høydesamling for OL 2016. Aktuelle utøvere i utholdenhetsidretter vil bli forespurt om å delta.

## Eksklusjonskriterier

- Allergier mot de aktuelle matvarer der det ikke er mulig å erstatte matvarer med liknende antioksidantrike produkter i samråd med deltakeren.
- Bruk av medisiner som kan påvirke treningen og prestasjonstester.
- Skader som kan påvirke trening og prestasjonstester.

## Rekruttering

Deltakere vil bli rekruttert fra potensielle OL 2016-utøvere i utholdenhetsidretter. Utøverne vil bli randomisert til en intervensjonsgruppe eller kontrollgruppe stratifisert for idrett og kjønn.

## Styrkeberegninger

Basert på en effektstørrelse på 1.0, med to-sidig signifikansnivå på 5% og 80% power trenger vi 17 idrettsutøvere i hver gruppe. Estimert endring i effektstørrelse er basert på variasjonen i prestasjonstestene i 2000 m roing på roergometer der varaiasjonen fra dag til dag er på 1.3 % (SD) under ellers like forhold. En endring i prestasjon på 1.3% tilsvarer ca. 5 sekunder forbedring på 2000 m roing; en bedring som kan skille medaljevinnere i et OL.  Samme relative endringer er ventet i prestasjon på de andre idrettene representert i forsøket. Dersom vi beregner en dropout % på 10 vil vi trenge 19 i hver gruppe for å påvise signifikante forskjeller mellom gruppene. Vi vil invitere alle aktuelle utøvere til å delta (n=45) fordi vi regner med at 85% samtykker til å delta.

## Tidsakse

Deltakerne vil bli rekruttert fire uker før høydeoppholdet i slutten av september 2015. Intervensjonen og høydeoppholdet i Spania vil avsluttes 11.november 2015. Alle deltakere vil gjennomgå en generell medisinsk undersøkelse og få kartlagt kliniske parametere tre uker i forkant av høydeopphold. I tillegg kartlegges hemoglobinmasse og kroppssammensetning én uke i forkant og i etterkant av høydeoppholdet. Det vil bli foretatt repeterte registreringer av kosthold, antropometri og prestasjon, samt blodprøvetaking, før, under og etter høydeoppholdet (Figur 1).

Figur 1. Tidslinje av studieforløpet.
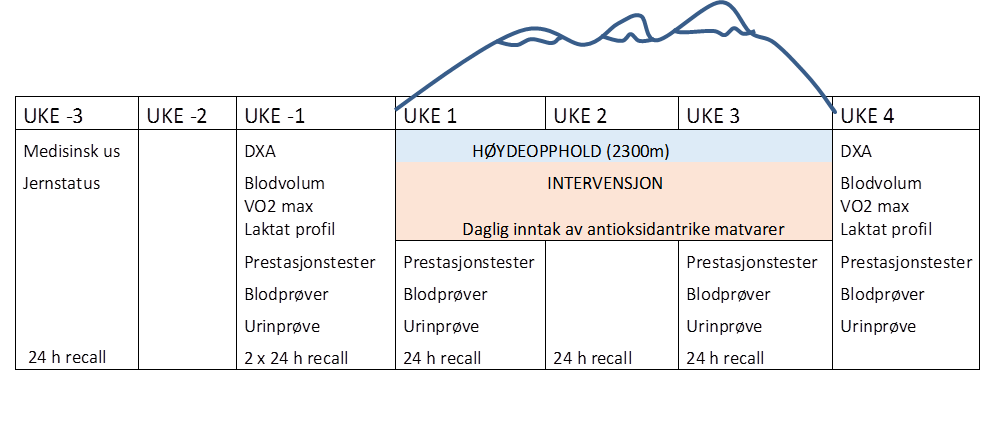


# Metoder

## Intervensjonen

Intervensjonens hensikt er å optimalisere mellommåltider hos idrettsutøvere under høydeopphold med hensyn på inntak av antioksidantrike matvarer. Intervensjonsgruppen vil motta matvarer med høyt innhold av antioksidanter (bl.a. bær, nøtter, mørk sjokolade, grønnsaker og frukt som har et naturlig høyt nivå av antioksidanter (Carlsen, Halvorsen et al. 2010). Kontrollgruppen vil få matvarer som vanligvis inntas som mellommåltider under høydeopphold. Mellommåltidene til gruppene vil bli matchet med hensyn til kaloriinnhold (ca 1000 kcal), og vil bli utdelt en gang om dagen til den enkelte deltaker. Utøverne i begge grupper oppfordres ellers til å holde stabil kroppsvekt (unngå avvik i kroppsvekt > 2 %) gjennom hele høydeoppholdet.

Mellommåltider intervensjonsgruppen (daglige)

Målet er å doble antioksidantinntaket til utøverne i intervensjonsgruppen ved å tilby ulike antioksidantrike mellommåltider. Intervensjonen som er beskrevet nedenfor er et eksempel. Andre vanlige antioksidantrike matvarer kan også inngå.

- 3 stk frukt/bær/grønnsak smoothie valgt fra følgende produkter (450 kcal):
- Bama, Turn 250 ml (71 kcal/100ml): 44 % druejuice, 30 % bananpuré, 17 % granateplejuice og 9 % solbærjuice
- Bama, Slide 250 ml (47 kcal/100ml): 38 % eplejuice, 20 % gulrotpuré, 20 % mangopuré 20 %, 17 % eplepuré og 5% pasjonsfruktpuré
- Bama, Spin 250 ml (62 kcal/100ml): 38 % eplejuice, 21 % rød druejuice, 20 % bananpuré, 8 % blåbærpuré, 8 % solbærjuice og 5 % eplepuré
- 1 dl (40 g) valnøtter (270 kcal)
- 1 dl (50g) blanding av tørket frukt og bær; tranebær, gojibær, aprikos (Rema1000 og Bama) (140 kcal)
- 40g mørk sjokolade (70%), utvalg av Freia Premium sjokolader i smaksvarianter; vanlig, pekan og havsalt, appelsin, mandel, hasselnøtt (240kcal)

Kontrollgruppen vil innta daglig:

- 220 ml TINE milkshake i smaksvarianter; jordbær og banan eller kakao (160 kcal)
- 330ml TINE YT restitusjonsdrikk i smaksvarianter; jordbær og banan eller kakao (220 kcal)
- 4 Bixit kjeks (60 g), eller annen type søte kjeks uten fyll (290 kcal)
- 1 dl (30g) Ritz salte kjeks /saltstenger (120 kcal)
- 50g Freia hvit sjokolade (270 kcal)

***Antropometri***

Standardiserte DXAmåling (dual energy x-ray; iDXA; GE Healthcare) tas før og etter treningsleir for å måle muskelmasse (LBM), fettmasse (FM) og bentetthet (BMD). Kroppsvekt med minst mulig bekledning før frokost registreres daglig og utøverne oppfordres til å holde stabil kroppsvekt (unngå avvik i kroppsvekt > 2%) gjennom hele høydeleiren.

***Kostregistreringsmetoder***

Det blir gjennomført fire 24 h recalls i en intervjusetting før høydeoppholdet, og tre ganger under høydeoppholdet av samme ernæringsfysiolog. 24 h recalls vil være separert med minst 2 dager. Intervjuguide for 24 h recall følger i eget vedlegg.

***Blodprøver***

Fastende blodprøver med venepunksjon samles før og etter høydesamling samt på to testdager i løpet av høydeoppholdet. I tillegg tas det ukentlige blodprøver fra fingertuppen under høydesamlingen. Det brukes Dried Blood Spot metode som muliggjør målinger av diverse biomarkører på en mindre invasiv måte. Laboratoriet ved UiO vil analysere blodprøvene mht biomarkører som f.eks. oksidativ skade, inflammasjonsstatus, antioksidantstatus og effekt på gen-ekspresjon i blodceller. Protokoll for blodprøvetaking og håndtering følger i eget vedlegg. Helblod gentranskripsjonsanalyser, low density gene arrays og/eller RT-PCR vil bli utført på blodceller som blir innsamlet ved de ulike tidspunktene.

***Blodvolums målinger***

Hemoglobinmasse, plasmavolum og totalt blodvolum vil bli estimert ved CO-rebreathing-metoden etter Burge og Skinner (Burge and Skinner 1995). Denne prosedyren vil gjennomføres to ganger før og etter intervensjonsperioden i høyden.

***Fysiologiske tester og prestasjonstester***

Deltakerne i denne studien er toppidrettsutøvere med tilknytting til Olympiatoppen. Alle utøverne har således erfaring med testene beskrevet under.

Prestasjonstesten og maksimalt oksygenopptak (VO2maks) vil kun testes ved Olympiatoppen (i lavlandet) før og etter intervensjonsperioden i høyden. Laktatprofiltesten (uten oksygenopptaksmålinger) vil også gjennomføres i høyden: 4-5 dager etter deltakerne kommer til høyden og 2-3 dager før hjemreise.

***Laktatprofiltest***

Deltakerne vil i et idrettsspesifikt ergometer/element (sykkel-, ro- og padleapparat, eller vann) gjennomføre en laktatprofiltest. Dette gjennomføres etter standard prosedyre ved Olympiatoppen (Stoa, Storen et al. 2010). Etter 10 min oppvarming på 60-70 % av HF-maks, gjennomføres 4 til 6 intervalldrag på 5 minutters med økende intensitet. Testen avsluttes når blodlaktatverdien er 1,5 mmol høyere enn laktatverdien på de to første intervalldragene. Mellom hvert intervalldrag er det en pause på 30 sekunder.

Laktat måles i helblod tatt ved fingerstikk. Fingertuppen vaskes og tørkes før hvert stikk. Oksygenopptak^[[1]](#footnote-1)^ (se under) og hjertefrekvens registreres underveis på hvert intervalldrag.

***Maksimal oksygenopp*t*ak (VO2maks)***

I forlengelsen av laktatprofiltesten gjennomføres det en VO2maks-test etter standard prosedyre ved Olympiatoppen (Tonnessen, Haugen et al. 2015). Testen starter på ett intensitetsnivå under anaerob terskel, og intensiteten økes hvert minutt. Testen gjennomføres til utmattelse, og varer typisk i 4-6 min. Svømmere vil måle VO2maks ved løping i motbakke.

***Prestasjonstest***

I et idrettsspesifikt ergometer/element vil deltakerne gjennomføre distanse test, der målet er å produsere så høy gjennomsnitts-watt/hastighet som mulig. Varigheten vil variere fra ca 2 min til 15 min avhengig av deltakerens idrettsgren.

- Sykling: 10 000 m på sykkelrulle
- Roing: 2000 m i Concept2-roapparat
- Padling: 1000 m Concept2-padleapparat
- Svømming: 200 m i vann (egen svømmeart)

Hjertefrekvens måles underveis, mens laktat måles 1 min etter avsluttet test.

***Dokumentasjon av trening***

Før, under og etter høydeoppholdet registrerer utøverne utført trening i Olympiatoppens treningsdagbok. I treningsdagboken registreres varigheten og intensiteten på treningen, samt treningsform og bevegelsesform. Treningen dokumenteres på samme måte som tidligere beskrevet beskrevet (Tonnessen, Sylta et al. 2014).

Hjertefrekvens måles underveis, mens laktat måles 1 min etter avsluttet test.

## Oppmøteoversikt

Visitt 1: Målingene foregår på helseavdeling på Olympiatoppen i Oslo.

- Generell medisinsk undersøkelse
- Blodprøver (venøs)
- Måling av hemoglobinmasse
- 24 timers recall (se vedlegg)

Visitt 2: Målingene foregår på helseavdeling og testlab på Olympiatoppen i Oslo.

- Fastende DXA måling (på NIH)
- Måling av hemoglobinmasse
- 24 h recall
- Laktatprofil
- VO2maks

Visitt 3: Målingene foregår på helseavdeling og testlab på Olympiatoppen i Oslo.

- Fastende blodprøver (venøs og fingertupp) og urinprøve
- Blodprøver etter frokost (venøs og fingertupp)
- Prestasjonstester
- Blodprøver etter prestasjonstester (venøs og fingertupp)
- 24 h recall

Visitt 4: Målingene foregår i Centro de alto rendimento i Sierra Nevada, Spania.

- Fastende blodprøver (venøs og fingertupp) og urinprøve
- Blodprøver etter frokost (venøs og fingertupp)
- Laktatprofil
- Blodprøver etter laktatprofil (venøs og fingertupp)
- 24 h recall

Visitt 5: Målingene foregår i Centro de alto rendimento i Sierra Nevada, Spania.

- Fastende blodprøver (venøs og fingertupp) og urinprøve
- Blodprøver etter frokost, rett før laktatprofil (venøs og fingertupp)
- Laktatprofil
- Blodprøver etter laktatprofil (venøs og fingertupp)
- 24 h recall

Visitt 6: Målingene foregår på helseavdeling og testlab på Olympiatoppen i Oslo.

- Fastende blodprøver (venøs og fingertupp) og urinprøve
- Måling av hemoglobinmasse
- Blodprøver etter frokost (venøs og fingertupp)
- Prestasjonstester
- Blodprøver etter prestasjonstester (venøs og fingertupp)

Visitt 7: Målingene foregår på helseavdeling og testlab på Olympiatoppen i Oslo

- Fastende DXA måling
- Måling av hemoglobinmasse
- 24 h recall
- Laktatprofil
- VO2maks

**Referanser**

Burge, C. M. and S. L. Skinner (1995). "Determination of hemoglobin mass and blood volume with CO: evaluation and application of a method." J Appl Physiol (1985) **79**(2): 623-631.

Bøhn, S. K., M. C. Myhrstad, M. Thoresen, M. Holden, A. Karlsen, S. H. Tunheim, I. Erlund, M. Svendsen, I. Seljeflot, J. O. Moskaug, A. K. Duttaroy, P. Laake, H. Arnesen, S. Tonstad, A. Collins, C. A. Drevon and R. Blomhoff (2010). "Blood cell gene expression associated with cellular stress defense is modulated by antioxidant-rich food in a randomised controlled clinical trial of male smokers." BMC Med **8**: 54.

Carlsen, K. H., E. Hem and T. Stensrud (2011). "Asthma in adolescent athletes." Br J Sports Med **45**(16): 1266-1271.

Carlsen, M. H., B. L. Halvorsen, K. Holte, S. K. Bohn, S. Dragland, L. Sampson, C. Willey, H. Senoo, Y. Umezono, C. Sanada, I. Barikmo, N. Berhe, W. C. Willett, K. M. Phillips, D. R. Jacobs, Jr. and R. Blomhoff (2010). "The total antioxidant content of more than 3100 foods, beverages, spices, herbs and supplements used worldwide." Nutr J **9**: 3.

Dean, B. J., P. Gettings, S. G. Dakin and A. J. Carr (2015). "Are inflammatory cells increased in painful human tendinopathy? A systematic review." Br J Sports Med.

Elkington, L. J., M. Gleeson, D. B. Pyne, R. Callister and L. G. Wood (2015). Inflammation and Immune Function: Can Antioxidants Help the Endurance Athlete? Antioxidants in Sport Nutrition. M. Lamprecht. Boca Raton FL, 2015 by Taylor & Francis Group, LLC.

Gomez-Cabrera, M. C., E. Domenech, M. Romagnoli, A. Arduini, C. Borras, F. V. Pallardo, J. Sastre and J. Vina (2008). "Oral administration of vitamin C decreases muscle mitochondrial biogenesis and hampers training-induced adaptations in endurance performance." Am J Clin Nutr **87**(1): 142-149.

Hu, F. B. (2003). "Plant-based foods and prevention of cardiovascular disease: an overview." Am J Clin Nutr **78**(3 Suppl): 544S-551S.

Knez, W. L., D. G. Jenkins and J. S. Coombes (2014). "The effect of an increased training volume on oxidative stress." Int J Sports Med **35**(1): 8-13.

Lewis, N. A., G. Howatson, K. Morton, J. Hill and C. R. Pedlar (2015). "Alterations in redox homeostasis in the elite endurance athlete." Sports Med **45**(3): 379-409.

Mazzeo, R. S. (2005). "Altitude, exercise and immune function." Exerc Immunol Rev **11**: 6-16.

Metz, L. N., R. Wustrack, A. F. Lovell and A. J. Sawyer (2012). "Infectious, inflammatory, and metabolic diseases affecting the athlete's spine." Clin Sports Med **31**(3): 535-567.

Paulsen, G., K. T. Cumming, G. Holden, J. Hallen, B. R. Ronnestad, O. Sveen, A. Skaug, I. Paur, N. E. Bastani, H. N. Ostgaard, C. Buer, M. Midttun, F. Freuchen, H. Wiig, E. T. Ulseth, I. Garthe, R. Blomhoff, H. B. Benestad and T. Raastad (2014). "Vitamin C and E supplementation hampers cellular adaptation to endurance training in humans: a double-blind, randomised, controlled trial." J Physiol **592**(Pt 8): 1887-1901.

Pialoux, V., R. Mounier, J. V. Brugniaux, E. Rock, A. Mazur, J. P. Richalet, P. Robach, J. Coudert and N. Fellmann (2009). "Thirteen days of "live high-train low" does not affect prooxidant/antioxidant balance in elite swimmers." Eur J Appl Physiol **106**(4): 517-524.

Pialoux, V., R. Mounier, E. Rock, A. Mazur, L. Schmitt, J. P. Richalet, P. Robach, J. Brugniaux, J. Coudert and N. Fellmann (2009). "Effects of the 'live high-train low' method on prooxidant/antioxidant balance on elite athletes." Eur J Clin Nutr **63**(6): 756-762.

Plunkett, B. A., R. Callister, T. A. Watson and M. L. Garg (2010). "Dietary antioxidant restriction affects the inflammatory response in athletes." Br J Nutr **103**(8): 1179-1184.

Powers, S. K., J. Duarte, A. N. Kavazis and E. E. Talbert (2010). "Reactive oxygen species are signalling molecules for skeletal muscle adaptation." Exp Physiol **95**(1): 1-9.

Powers, S. K. and M. J. Jackson (2008). "Exercise-induced oxidative stress: cellular mechanisms and impact on muscle force production." Physiol Rev **88**(4): 1243-1276.

Ristow, M., K. Zarse, A. Oberbach, N. Kloting, M. Birringer, M. Kiehntopf, M. Stumvoll, C. R. Kahn and M. Bluher (2009). "Antioxidants prevent health-promoting effects of physical exercise in humans." Proc Natl Acad Sci U S A **106**(21): 8665-8670.

Stoa, E. M., O. Storen, E. Enoksen and F. Ingjer (2010). "Percent utilization of VO2 max at 5-km competition velocity does not determine time performance at 5 km among elite distance runners." J Strength Cond Res **24**(5): 1340-1345.

Sugama, K., K. Suzuki, K. Yoshitani, K. Shiraishi, S. Miura, H. Yoshioka, Y. Mori and T. Kometani (2015). "Changes of thioredoxin, oxidative stress markers, inflammation and muscle/renal damage following intensive endurance exercise." Exerc Immunol Rev **21**: 130-142.

Tonnessen, E., T. A. Haugen, E. Hem, S. Leirstein and S. Seiler (2015). "Maximal Aerobic Capacity in the Winter Olympic Endurance Disciplines: Olympic Medal Benchmarks for the Time Period 1990-2013." Int J Sports Physiol Perform.

Tonnessen, E., O. Sylta, T. A. Haugen, E. Hem, I. S. Svendsen and S. Seiler (2014). "The road to gold: training and peaking characteristics in the year prior to a gold medal endurance performance." PLoS One **9**(7): e101796.

Turagam, M. K., P. Velagapudi and A. G. Kocheril (2012). "Atrial fibrillation in athletes." Am J Cardiol **109**(2): 296-302.

Watson, T. A., R. Callister, R. D. Taylor, D. W. Sibbritt, L. K. MacDonald-Wicks and M. L. Garg (2005). "Antioxidant restriction and oxidative stress in short-duration exhaustive exercise." Med Sci Sports Exerc **37**(1): 63-71.

1. Oksygenopptak vil ikke måles hos svømmere. [↑](#footnote-ref-1)
